# High-Level Production of Recombinant Snowdrop Lectin in Sugarcane and Energy Cane

**DOI:** 10.3389/fbioe.2020.00977

**Published:** 2020-08-18

**Authors:** Carmen S. Padilla, Mona B. Damaj, Zhong-Nan Yang, Joe Molina, Brian R. Berquist, Earl L. White, Nora Solís-Gracia, Jorge Da Silva, Kranthi K. Mandadi

**Affiliations:** ^1^Texas A&M AgriLife Research and Extension Center, Weslaco, TX, United States; ^2^Institute for Plant Gene Function, Department of Biology, Shanghai Normal University, Shanghai, China; ^3^iBio, Bryan, TX, United States; ^4^MDx BioAnalytical Laboratory, Inc., College Station, TX, United States; ^5^Department of Soil and Crop Sciences, Texas A&M University, College Station, TX, United States; ^6^Department of Plant Pathology and Microbiology, Texas A&M University, College Station, TX, United States

**Keywords:** therapeutic protein, recombinant protein, snowdrop-bulb lectin, *Galanthus nivalis* agglutinin, promoter stacking, biofactory, *Saccharum* species

## Abstract

Sugarcane and energy cane (*Saccharum* spp. hybrids) are ideal for plant-based production of recombinant proteins because their high resource-use efficiency, rapid growth and efficient photosynthesis enable extensive biomass production and protein accumulation at a cost-effective scale. Here, we aimed to develop these species as efficient platforms to produce recombinant *Galanthus nivalis* L. (snowdrop) agglutinin (GNA), a monocot-bulb mannose-specific lectin with potent antiviral, antifungal and antitumor activities. Initially, GNA levels of 0.04% and 0.3% total soluble protein (TSP) (0.3 and 3.8 mg kg^–1^ tissue) were recovered from the culms and leaves, respectively, of sugarcane lines expressing recombinant *GNA* under the control of the constitutive maize *ubiquitin 1* (*Ubi*) promoter. Co-expression of recombinant GNA from stacked multiple promoters (*pUbi* and culm-regulated promoters from sugarcane *dirigent5-1* and *Sugarcane bacilliform virus*) on separate expression vectors increased GNA yields up to 42.3-fold (1.8% TSP or 12.7 mg kg^–1^ tissue) and 7.7-fold (2.3% TSP or 29.3 mg kg^–1^ tissue) in sugarcane and energy cane lines, respectively. Moreover, inducing promoter activity in the leaves of GNA transgenic lines with stress-regulated hormones increased GNA accumulation to 2.7% TSP (37.2 mg kg^–1^ tissue). Purification by mannose-agarose affinity chromatography yielded a functional sugarcane recombinant GNA with binding substrate specificity similar to that of native snowdrop-bulb GNA, as shown by enzyme-linked lectin and mannose-binding inhibition assays. The size and molecular weight of recombinant GNA were identical to those of native GNA, as determined by size-exclusion chromatography and MALDI-TOF mass spectrometry. This work demonstrates the feasibility of producing recombinant GNA at high levels in *Saccharum* species, with the long-term goal of using it as a broad-spectrum antiviral carrier molecule for hemopurifiers and in related therapeutic applications.

## Introduction

The production of recombinant therapeutic proteins on a large scale is a fast-growing sector of biopharmaceutical research and industry. Many proteins are currently produced using conventional cell culture-based systems, including those using mammals and microbes (Alqazlan, 2014). However, the need for a platform that offers low production costs, safety and high scalability has led to the use of plants as biofactories (Williams et al., 2014; Chen and Davis, 2016).

Sugarcane and energy cane (*Saccharum* spp. hybrids) are ideal platforms for the production of native and recombinant protein-based therapeutics at commercial levels, due to their high resource-use efficiencies, rapid growth, efficient photosynthesis and high biomass production capacity, with potential yields of up to 49 tons of dry biomass per hectare per annum (Ando et al., 2011; Matsuoka et al., 2014). In general, sugarcane offers a high level of transgene containment (Altpeter and Oraby, 2010). Sugarcane/energy cane are primarily propagated by vegetative means and natural reproductive propagation is rare in many temperate and subtropical regions due to its photoperiod sensitivity, thus limiting transgene flow by pollen. Furthermore, many of the commercial sugarcane or energy cane varieties do not produce viable pollen or seeds under typical field conditions. Sugarcane has been tested as a potential biofactory for the production of recombinant proteins such as the human cytokine granulocyte-macrophage colony-stimulating factor GM-CSF (Wang M.L. et al., 2005), canecystatins (cysteine protease inhibitors) (Gianotti et al., 2006; Ribeiro et al., 2008; Henrique-Silva and Soares-Costa, 2012) and the cellulolytic enzymes endoglucanase and cellobiohydrolases I and II (Harrison et al., 2011; Harrison et al., 2014). The accumulation level of these recombinant proteins ranged from 0.02% to 2.0% of the total soluble protein (TSP) in leaves. By contrast, energy cane has not been studied for its use as a biofactory for recombinant therapeutic proteins.

Lectins related to *Galanthus nivalis* L. (snowdrop) agglutinin (GNA), which is tetrameric, represent a superfamily of strictly 2-D-mannose-binding-specific lectins from bulbs that is widespread among monocots. The subunits of all GNA-related lectins share a similar three-dimensional structure with GNA, despite differences in their amino-acid sequences (Barre et al., 1996). The clinical application of GNA-related plant-bulb lectins is an ongoing area of research due to their important antitumor, antifungal and antiviral activities (Damodaran et al., 2008; Li et al., 2009; Liu et al., 2009; Beyene et al., 2011; Wu and Bao, 2013; Akkouh et al., 2015). The first GNA-related lectin, termed GNA, was isolated from snowdrop bulbs and is composed of four identical subunits of about 12.0 kDa (Van Damme et al., 1987). The GNA lectin has exclusive binding specificity for mannose and has been characterized in particular for its potent inhibition of retroviruses (Akkouh et al., 2015). GNA inhibits hepatitis C virus infection of serum in a dose-dependent manner by binding N-linked glycans located at the top of the viral envelope (Ashfaq et al., 2011). Additionally, GNA selectively inhibits several varieties of immunodeficiency type 1 and 2 viruses in different cell types (Balzarini et al., 2004) and prevents cell-cell fusion in cells expressing HIV viral envelope glycoproteins and T cells (CD4^+^) (Yee et al., 2011).

GNAs and other plant lectins have been produced as recombinant and heterologous proteins as an alternative to the native sources. The main expression systems were using bacteria (*Escherichia coli*) and yeast (*Pichia pastoris*) and to some extent plant and mammalian cells (Oliveira et al., 2013; Martínez-Alarcón et al., 2018). Production in microbes results in good yields, however the recombinant lectins are often insoluble or not properly processed, requiring further downstream processing like refolding, extraction from inclusion bodies and post-translational modifications such as glycosylation. In this context, plant-based systems are relatively closer to native snowdrop lily conditions when compared to other prokaryotic or yeast systems. Among the plant-based systems, GNA was produced in transgenic rice, maize and sugarcane, mainly in leaves with yields reaching approximately 0.1–0.5% TSP, 0.28% TSP and 0.89% TSP, respectively (Rao et al., 1998; Tinjuangjun et al., 2000; Tang et al., 2001; Setamou et al., 2002; Nagadhara et al., 2003; Wang Z. et al., 2005).

In this work, we demonstrate the feasibility of sugarcane and energy cane as platforms for the efficient production of recombinant snowdrop GNA protein at high levels. In transgenic sugarcane, GNA yields using a constitutive single promoter (maize *ubiquitin 1*) reached 0.04% TSP and 0.3% TSP in culms and leaves, respectively. GNA accumulation was increased further to 1.8% TSP and 2.3% TSP in culms and leaves, respectively, of sugarcane and energy cane by co-expressing recombinant *GNA* using a stack of three different constitutive and culm-regulated promoters and combinatorial plant transformation. The highest GNA level of up to 2.7% TSP was achieved by inducing the promoter activity of GNA transgenic lines with stress-regulated hormones. Mannose-agarose affinity chromatography purification allowed the recovery of a functional GNA that retained binding specificity to anti-GNA antibody and the mannose substrate in a manner similar to the native snowdrop-bulb GNA.

## Materials and Methods

### Expression Vectors

A series of *GNA* expression vectors was constructed using the full-length cDNA (570 bp) that encodes *G. nivalis* L. (snowdrop) lectin, LECGNA2 (GenBank Accession Number M55556, Protein Data Bank accession number AAA33346). The full-length *GNA* cDNA was obtained from Van Damme et al. (1991) in the pT7T3-18U vector (Addgene, Watertown, MA, United States).

A recombinant GNA, GNA_109_ (*G. nivalis* lectin LECGNA2 with four additional amino acids, Thr_106_ His_107_ Thr_108_ Gly_109_, at the C-terminus of the mature protein), was synthesized (GenScript United States, Inc., Piscataway, NJ, United States) and used as a control due to its known binding affinity to mannan (Raemaekers, 2000).

### Single-Terminator Vectors

The *GNA* expression vector *pUbi*-*GNA*-*NOST*/pUC18, which contains the constitutive maize *ubiquitin 1* promoter (*pUbi*), was generated (Christensen et al., 1992; Christensen and Quail, 1996). The *GNA* full-length cDNA (570 bp) was excised from *GNA*/pT7T3-18U with *Eco*RI, filled in and cloned into *Sal*I-digested/filled-in pUNos_C1 (a gift from Jane Glazebrook and Fumi Katagiri; Addgene plasmid # 33297), which is pUC18 with *pUbi* and the terminator from the *Agrobacterium tumefaciens* nopaline synthase gene (*NOST*).

### Double-Terminator Vectors

Four *GNA* expression vectors with a double terminator were generated. The first vector, *pUbi-GNA-35STNOST/*pZero2, was constructed with *pUbi* (1,977 bp) and the double terminators *NOST* (253 bp) and the 197-bp *Cauliflower mosaic virus* 35S polyadenylation signal (*35ST*) (Beyene et al., 2011). The *GNA* fragment (570 bp) was amplified from *pUbi-GNA-NOST*/pUC18, *Bam*HI restriction sites were added using the primers GNA-1F 5′-GGATCCCAACTACAAGTTACAAAATGGCTA-3′ and GNA-570R 5′-GGATCCCGCGACGAGGTCGATTATCTCAAA-3′ and the fragment was fused to the *Bam*HI-digested *pUbi*-*BvLz*_*m*_-*35STNOST*/pZero2 vector (Damaj and Mirkov, 2019), to replace the *BvLz*_*m*_ fragment. The second vector, *pSHEF1*α-*GNA*-*35STNOST*/pNEB193, was assembled as follows: the *Xba*I/*Bbs*I-digested/filled-in *pUbi-GNA*-*35STNOST/* pZero2 *GNA-35STNOST* fragment was fused to *Bam*HI-digested/filled-in pNEB193 (New England BioLabs, Ipswich, MA). The *pSHEF1*α fragment (1,959 bp), excised from *pSHEF1*α/pSK^+^ (Yang et al., 2003) with *Eco*RI and filled in, was fused to *GNA* in *GNA*-*35STNOST*/pNEB193 following *Asc*I digestion and filling in.

The two remaining *GNA* expression vectors containing a double terminator were generated to include the culm-regulated promoters from *Sugarcane bacilliform virus* (*pSCBV21*) (Gao et al., 2017) and from the *Saccharum* spp. hybrid *dirigent5-1* gene (*pSHDIR5-1*) (Damaj et al., 2010; Damaj and Mirkov, 2017). The *pSCBV21-GNA-35STNOST*/pGEMT-T Easy vector was created by cloning the *GNA* fragment (570 bp), amplified from *pUbi-GNA-NOST*/pUC18 with added *Bam*HI restriction sites (using the primers GNA-1F and GNA-570R), into the *Bam*HI-digested *pSCVB21* (1,816 bp)-*BvLz*_*m*_-*35STNOST*/pGEM-T Easy vector (Damaj and Mirkov, 2019), thereby replacing the *BvLz*_*m*_ fragment. To construct the *pSHDIR5-1-GNA-35STNOST*/pGEM-T Easy vector, the *pSHDIR5-1* fragment (4,710 bp), excised from *pSHDIR5-1-GUS-NOST*/pUC19 (Damaj and Mirkov, 2017) with *Hin*dIII/*Spe*I and filled in, was fused to *GNA* in *Sac*II/*Spe*I-digested and filled-in *pSCBV21-GNA-35STNOST*/pGEMT-T Easy vector, thereby replacing pSCBV21.

All DNA cloning steps were carried out as previously described (Sambrook et al., 1989). The filling in of ends of digested DNA fragments and the dephosphorylation of vectors were performed using T4 DNA polymerase (New England BioLabs, Ipswich, MA, United States) and Antarctic Phosphatase (New England BioLabs), respectively. The PCR amplification was performed using Platinum^TM^ PCR SuperMix High Fidelity (Invitrogen, ThermoFisher Scientific, Waltham, MA, United States).

### Plant Transformation

The tops of field-grown sugarcane (*Saccharum* spp. hybrids) commercial varieties CP72-1210, CP89-2143, TCP87-3388, TCP89-3505 and TCP98-4454, and energy cane varieties TCP10-4928 and Ho02-113 were collected during the growing season, and leaf-roll discs were prepared for stable transformation mainly as previously described (Ramasamy et al., 2018). Briefly, leaf blades and sheaths were removed down to leaf 1 (the topmost visible dewlap leaf), and the upper 20–30 cm portion of the shoot (leaf-roll culm) was surface-sterilized in 70% (v/v) ethanol for 20 min. Immature leaf rolls close to the apical meristem were sliced transversely into 1-mm thick sections and cultured on MS3 medium (Murashige and Skoog medium [MS] with 3 mg L^–1^ 2,4-dichlorophenoxyacetic acid [2,4-D]) for 30–35 days (for the generation of embryogenic calli) (Murashige and Skoog, 1962) or on MS0.6 medium (MS with 0.6 mg L^–1^ 2,4-D) for 7-10 days (for the generation of embryogenic leaf-roll discs) (Snyman et al., 2006). Embryogenic calli and leaf-roll discs were preconditioned on MS3- and MS0.6-osmoticum supplemented with 0.2 M D-mannitol and 0.2 M D-sorbitol, respectively, for 4 h before and after DNA particle bombardment. The DNA bombardment was performed according to Ramasamy et al. (2018). Briefly, gold particles (0.3 μm, Crescent Chemical Company, Islandia, NY, United States) (1 mg) were coated separately with 1.0 μg plasmid DNA of different constructs in equimolar ratios together with the *pUbi:BAR*/pUC8 selectable marker plasmid, using 1 M calcium chloride and 14 mM spermidine. The DNA particle suspension (containing the selectable marker plasmid with one or more *GNA* plasmids) (4 μL, 0.5 μg DNA per bombardment) was placed at the center of a syringe filter and delivered into tissue by a particle inflow gun using a 26-inch Hg vacuum and 7-cm target distance. Bombarded embryogenic calli were maintained on MS3 for 10 days in the dark at 28°C for recovery. Shoot regeneration and root initiation were performed under bialaphos selection (3 mg L^–1^ for sugarcane and 1.5 mg L^–1^ for energy cane) as described previously (Gao et al., 2013; Ramasamy et al., 2018). Rooted plantlets were transferred to potting soil (Metro-Mix, Scotts, Hope, AR, United States) in pots and maintained in the greenhouse at 25–30°C during the day and 15–24°C at night with a light intensity of 1,200–1,600 μmol m^–1^ s^–1^ at midday.

### Transgenic Plant Screening

Integration and size of each *GNA* expression cassette in the single and multiple stacked promoter:*GNA* sugarcane lines were determined by Southern blotting and PCR analyses, respectively. Controls included vector-transformed lines and non-transformed plants (tissue culture-derived). Genomic DNA was isolated by grinding leaf tissues (3 g) collected from 3- to 4-month-old transgenic sugarcane plants in liquid nitrogen as previously reported (Tai and Tanksley, 1990; Chiong et al., 2017).

For Southern blot analysis, genomic DNA (15 μg per reaction) was digested overnight with *Sal*I, electrophoresed on 0.8% (w/v) agarose gels and transferred to nylon membranes (Amersham Hybond-XL, GE Healthcare Bio-Sciences Corp., Piscataway, NJ, United States) in 0.4 M sodium hydroxide (Sambrook and Russell, 2001). Pre-hybridization, hybridization, washing and detection of DNA gel blots were performed as described by Mangwende et al. (2009), using Church’s buffer. The *GNA*-specific probe (570 bp) was released from *pUbi:GNA*/pUC18 with *Pst*I. Probes were labeled with [α-^32^P]dCTP using the Random Primers DNA Labeling kit (Invitrogen, ThermoFisher Scientific) (Mangwende et al., 2009).

PCR was performed on a C1000 Touch^TM^ thermal cycler (Bio-Rad Laboratories, Inc., Hercules, CA) in a total reaction volume of 25.0 μL using 300.0 ng of DNA and AccuStartTM II PCR ToughMix^®^ (Quantabio, Beverly, MA, United States) according to the manufacturer’s instructions with the following conditions: 94°C for 3 min, 35 cycles each at 94°C for 30 s, 55-60°C for 30 s, and 72°C for 3-6 min. Primers encompassing the entire promoter:*GNA*-terminator cassette (Supplementary Table S1) were designed with Primer 3.0. All PCR amplicons were separated by electrophoresis on 0.7% agarose (w/v) gels stained with ethidium bromide. A “no DNA template” was included as a negative control and the transformation plasmid as a positive control for PCR.

### Plant Growth and Treatment Conditions

For experiments involving hormone induction, treatments were conducted by spraying 1-year-old transgenic plants of three single-promoter *pUbi-pSCBV21-pSHDIR5-1:GNA* lines and 12 triple-promoter *pUBD5:GNA* lines with 5 mM salicylic acid (SA) (S3007-500G; Sigma-Aldrich, St. Louis, MO) (in 0.05% [v/v] Tween-20 aqueous solution) in the greenhouse (28°C with 14 h-light/10 h-dark). Control plants were sprayed only with 0.05% Tween-20. Leaf samples were collected at 0 and 48 h following treatment. The plants were distributed randomly, with two plants per line grown in a 16-L pot, and four pots per line were selected for each time point.

### Bench-Scale Extraction of Recombinant GNA

For the bench-scale extraction of total soluble protein (TSP) from *GNA* transgenic sugarcane leaves, 200 mg of tissue was homogenized in 0.2 M sodium acetate and 0.2 M acetic acid (pH 5.2) buffer (600 μL) (1:3 tissue to buffer ratio) in 2-mL screw-cap microcentrifuge tubes for 30 s at 5,000 rpm with a Precellys 24 homogenizer (MO BIO Laboratories, Carlsbad, CA, United States) in the presence of a ceramic spherical bead (0.64 cm-diameter). The TSP supernatants were collected by centrifugation at 10,600 *g* twice for 10 min at 4°C and were stored at −80°C for analysis.

For the bench-scale extraction of TSP from *GNA* transgenic sugarcane culms, tissue was harvested, shredded with a garden shredder (MTD1400K, Yard machines, Home Depot, Weslaco, TX, United States) and frozen at −80°C. Frozen tissue (25 g) was ground with a IKA^®^-WERKE M20 Universal Mill (Breisgau, Germany), mixed with 50 mL extraction buffer (0.1 M citric acid/0.2 M sodium acetate buffer, pH 4.0 or 0.1 M sodium acetate/0.2 M acetic acid with 1 mM EDTA and 0.05% [v/v] Tween 20, pH 5.2) (1:2 tissue to buffer ratio) in a WARING^®^ commercial blender (Model 7011HS) for 5 min and heated at 65°C for 15 min with occasional shaking. The TSP supernatants were collected by centrifugation at 12,000 *g* for 20 min and clarified by being passed through four layers of Miracloth (475855-1R, Millipore Sigma, Darmstadt, Germany). Clarified TSPs were precipitated with acetone and 0.1% [v/v] β-mercaptoethanol (1:5) at −20°C for 1 h. The TSP pellets were collected by centrifugation at 12,000 *g* for 20 min at 4°C and resuspended in 50 μL of 6 × sample buffer (0.38 M Tris–HCl, pH 6.8, 10% SDS, 30% [v/v] glycerol, 30% [v/v] β-mercaptoethanol and 0.2% [w/v] bromophenol blue). The GNA standard was prepared from pure *Galanthus nivalis* snowdrop-bulb lectin (L-7401-5; EY Laboratories, Inc., San Mateo, CA, United States).

### Semi-Quantitative Immunoblot Analysis

The TSPs extracted from *GNA* transgenic sugarcane leaves and culms were boiled for 5 min in 12 μL of 6 × sample buffer and were analyzed by SDS-PAGE in Novex^®^ NuPAGE 4–12% Bis-Tris gels (Invitrogen, ThermoFisher Scientific). The TSP content of leaf and culm extracts was determined using the Qubit fluorometer (ThermoFisher Scientific) and the Lowry method together with the DC Protein Assay kit (Bio-Rad Laboratories Ltd., Hercules, CA, United States), respectively.

The TSPs were transferred onto NitroBind nitrocellulose membranes (0.22 μ) (GE Water and Process Technologies, Boulder, CO, United States) using a Mini Trans-Blot Electrophoretic Transfer Cell (Bio-Rad Laboratories Ltd.). Blocking of non-specific sites was performed with 5% [w/v] skim milk in 1 × Tris buffered saline (TBS)-Tween solution (TBST) (25 mM Tris–HCl, pH 7.8, 190 mM sodium chloride (NaCl) and 0.1% [v/v] Tween-20) for 1 h. Incubation with the polyclonal anti-GNA antibody (produced in rabbits; Research Genetics Inc., Huntsville, AL, United States) was performed for 1 h at a concentration of 1:1,000 in blocking solution (3% [w/v] skim milk, 1 × TBS), followed by three washes with 1 × TBST (1 × TBS with 0.05% [v/v] Tween 20). Samples were incubated with the peroxidase-linked goat anti-mouse secondary antibody (A6154, Sigma-Aldrich) (1:2,000 in 3% [w/v] skim milk, 1 × TBS blocking solution) for 2 h and then washed three times with 1 × TBST. Signals were detected by incubation with 4-chloro-1-napththol (Sigma-Aldrich) and hydrogen peroxide in 1 × TBS. Immunoblots were quantified using ImageJ software (https:imagej.nih.gov/ij/download.html) by comparing sample band intensity with that of the GNA standard (pure GNA protein) present on the same membrane.

### Affinity Chromatography Purification

A pool of leaves (892.0 g) from three representative single-promoter *pUbi:GNA* lines and four triple-promoter:*GNA* lines was mixed with extraction buffer (50 mM sodium phosphate, 150 mM NaCl, 5 mM EDTA, pH 8.0, 60 mM freshly prepared ascorbic acid and 1 mM phenylmethylsulfonyl fluoride) and was mechanically homogenized in batches. A final ratio of buffer to biomass of 6:1 (v/w) was used. The homogenate was clarified by centrifugation, gravity filtration and depth filtration. The clarified extract was concentrated 20-fold by tangential flow filtration (TFF) using a 5.0 kDa MWCO TFF cassette (Millipore Sigma, Danvers, MA, United States). Because the aim was to generate only sufficient material for analytical characterization, mannose-agarose affinity chromatography was employed to capture GNA (mannose agarose has a 45 mg mL^–1^ binding capacity for GNA). A 10 mL (5 cm) mannose agarose (M6400-10ML; Millipore Sigma) column (with the capacity to bind approximately 90 mg GNA) column was equilibrated with five column volumes (CVs) of 1 × phosphate buffered saline (PBS). The clarified extract was loaded at a flow velocity of 75 cm h^–1^ (a 4-min residence time). The column was washed with 20 CVs of 1 × PBS, and elution was performed with 10 CVs of 1 × PBS and 0.5 M mannose. The mannose-bound elution fractions, monitored by absorbance at 280 nm, were pooled, concentrated by TFF using a 5.0-kDa-MWCO membrane (Millipore Sigma) and diafiltered with 10 volumes of 1 × PBS to remove mannose.

### Size-Exclusion Chromatography

The mannose-agarose purified and concentrated sugarcane recombinant GNA was subjected to size-exclusion (SE) chromatography using a TSKgel G3000SW xL (Tosoh Bioscience, King of Prussia, PA, United States), 7.8 mm × 30 cm, 5 μm column and an HP 1100 Series HPLC system with a diode array detector (Agilent Technologies, Inc., Santa Clara, CA, United States). The mobile phase for SEC consisted of 50 mM sodium phosphate (monobasic, monohydrate)/sodium phosphate (dibasic, anhydrate) and 0.3 M NaCl, pH 7.0. An SE chromatography protein mix standard consisting of thyroglobulin (0.5 mg mL^–1^), bovine serum albumin (1 mg mL^–1^), ovalbumin (1 mg mL^–1^), α-lactalbumin (1 mg mL^–1^) and aprotinin (0.4 mg mL^–1^) with molecular weights of 675, 66.5, 45, 14.2 and 6.5 kDa, respectively, was used to determine the molecular weight of the sample chromatographic peaks. The protein mix molecular weight standard was prepared prior to sample analysis and 20 μL was injected into the column. Mobile phase buffer was used for blank injections. The column was equilibrated with the mobile phase before sample analysis. The GNA samples were separated on the column at a flow rate of 1.0 mL min^–1^ for a total run time of 15 min.

Data were analyzed using ChemStation Data Analysis software (Agilent Technologies, A.01.04 025) (Agilent, Santa Clara, CA, United States). Data were recorded at 220 nm and 280 nm, the UV signal of each GNA sample was integrated, and the relative percentage of each peak detected was determined. All peaks with a percent relative abundance ≥ 0.1% were considered for quantification.

### MALDI-TOF Mass Spectrometry

Following mannose-agarose purification and concentration, the molecular weight of the sugarcane recombinant GNA was analyzed under non-reducing conditions using matrix-assisted laser desorption ionization time-of-flight mass spectrometry (MALDI-TOF MS). The sinapinic acid (SiA) matrix was prepared at 10 mg mL^–1^ in 30:70 acetonitrile:0.1% trifluoroacetic acid:water (TFA:H_2_O). GNA samples were mixed 1:9 in a 0.5 mL Eppendorf^®^ centrifuge tube, and 1.5 μL of each mixture was spotted onto a MALDI plate.

The samples were co-crystalized with the matrix under a gentle flow of air prior to analysis (about 2–3 min). The plate was then loaded into the MALDI-TOF mass spectrometer (Applied Biosystems, Waltham, MA, United States) and acquisitions were performed manually. The instrument was operated in linear delayed positive ion mode with an accelerating voltage of 25 kV, the grid set to 66–74% and a delay time of 200 ns. The Shots/Spectrum was set to 200 with a mass range of 4,500–50,000 Da and a low mass gate of 4,000 Da. The SiA matrix was selected to acquire the calibration and data file spectra. The initial laser power was set to 1800 for calibration and was adjusted as necessary during spectrum acquisition. A one-point calibration curve was generated using the mean mass peak of myoglobin (16,952.5 Da) in the standard test mix and was used to analyze the samples. The calibration file was opened and entered in Voyager with Data Explorer^TM^ software for subsequent data acquisition and processing.

### Enzyme-Linked Lectin Assay

The binding efficiency of GNA to mannose was determined using the enzyme-linked lectin assay (ELLA), which allows lectin-mannose interactions to be analyzed in a standard microtiter plate format. The ELLA requires the immobilization of mannan onto the surface of an ELISA plate. Mannan derived from *Saccharomyces cerevisiae* was used, which has a short peptide tail and binds well to the plate.

As a coating substrate, mannan was immobilized onto an ELISA plate (96-well plate; ThermoFisher 62409-024) at 1 μg per well (using 100 μL per well of a pre-prepared stock solution of 10 μg mL^–1^ in 1 × PBS) with incubation overnight at 4°C. Mannan-coated plates were washed twice with PBST (1 × PBS, 0.2% [v/v] Tween 20) and were blocked with 150 μL of 1 × carbo-free blocker solution (Vector Laboratories, Inc. [Vector Labs]; Burlingame, CA, United States) for 2 h at 28°C. All subsequent steps were performed at 28°C. The wells were washed twice with 1 × PBST. Snowdrop-bulb native GNA (Vector Labs) was used to create a standard curve starting from 25 ng mL^–1^, with seven two-fold serial dilutions. Different dilutions of the purified GNA sample prepared in 0.1% carbo-free blocker solution were added to the ELISA plate and incubated for 1 h. Unbound lectin was removed by washing each well twice with PBST. The plates were then incubated with goat anti-GNA polyclonal antibody (100 μL per well) (1:1,000 in 0.1 × blocking solution) for 1 h at 28°C. After two washes with PBST, the plates were incubated for 1 h at 28°C with anti-goat IgG (whole molecule)-alkaline phosphatase antibody produced in rabbit (100 μL per well) (1:1,000 in 0.1 × blocking solution). Plates were subsequently washed twice with PBST and then once with 1 × PBS. Plates containing alkaline phosphatase yellow substrate (100 μL per well) were incubated in the dark at 28°C for 15 min. The reaction was stopped with 3 N sodium hydroxide (25 μL per well), and absorbance was measured at 405 nm using the 96-well Synergy H1 5.1 Hybrid Multi-Mode plate reader (BioTeck, Winnoski, VT, United States).

Inhibition assays were performed with a constant concentration (100 ng mL^–1^) of recombinant GNA samples pre-incubated for 1 h with two-fold serial dilutions (3.9, 7.8, 15.6, 31.3, 62.5, 125 and 250 mM) of methyl α-D-mannoside (Sigma-Aldrich) (100 mg mL^–1^ stock; MW 194), a competitive inhibitor of mannan binding. Samples were added to the plate that was pre-blocked with carbo-free blocker, and the ELLA protocol was performed as described above. The recombinant GNA concentration was estimated based on a standard curve of different concentrations of snowdrop-bulb native GNA (Vector Labs).

## Results and Discussion

### Recombinant Snowdrop-Bulb GNA Is Synthesized in Transgenic Sugarcane

**FIGURE 1:**
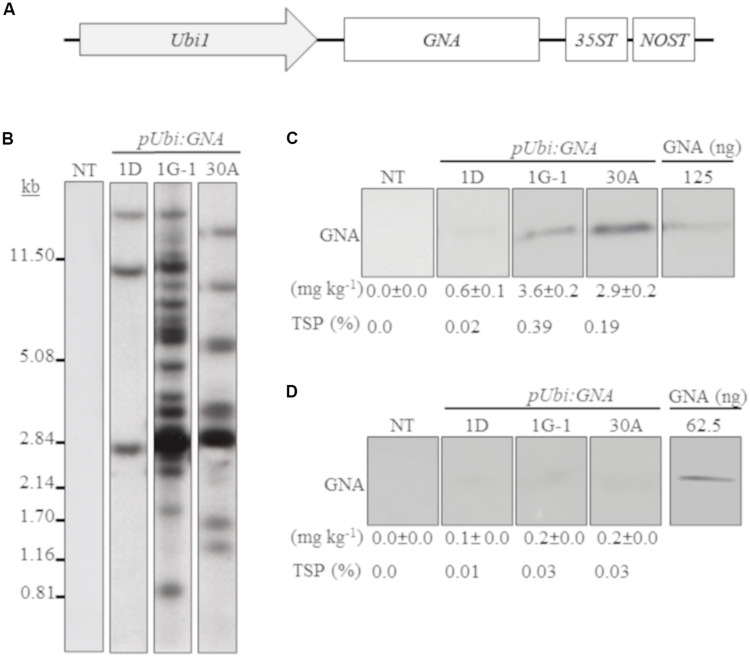
Molecular characterization of single-promoter:*GNA*-expressing lines. **(A)** Design of the expression vector with the constitutive maize *ubiquitin 1* promoter (*Ubi1*; *pUbi*), *GNA* and double-terminator *35ST* (from *Cauliflower mosaic virus* 35S RNA) and *NOST* (from *Agrobacterium tumefaciens* nopaline synthase). Vector assembly and cloning sites are described under ‘MATERIALS AND METHODS.’ **(B)** Stable integration of recombinant *GNA* in three representative sugarcane *pUbi:GNA* lines as detected by Southern blot analysis. DNA gel blots were hybridized to a probe corresponding to the *GNA* coding region. GNA accumulation in sugarcane leaves **(C)** and culms **(D)** of three representative sugarcane *pUbi:GNA* lines as assessed by semi-quantitative immunoblot analysis of total soluble proteins (TSP), using a polyclonal anti-GNA antibody. Molecular weight of GNA is ∼12 kDa. All immunoblots were loaded with an equal amount of TSP (60 μg) per lane, and 62.5 or 125 ng of snowdrop-bulb native GNA as a standard. The GNA yield (mg kg^–1^ of tissue) in clarified juice extract is indicated. Yield values represent two biological replicates and are reported with the standard error. NT: non-transformed (tissue culture derived) plant. The full-length uncropped DNA autoradiograms and TSP immunoblots are displayed in Supplementary Figures S1–S4, respectively.

We generated several independent transgenic sugarcane lines that expressed *GNA* from the single *Ubi* promoter and *35STNOST* double terminator (*pUbi:GNA-35STNOST*) (Figure 1A) and confirmed their identity by Southern blot analysis. These consisted of eight lines (20 plants) for TCP87-3388, nine lines (12 plants) for TCP89-3505, three lines (60 plants) for TCP98-4454 and seven lines (23 plants) for CP72-1210. Southern blot analysis revealed that the majority of the *pUbi:GNA* lines exhibited a simple integration pattern (two to five integration events) (Figure 1B; representative *pUbi:GNA* line 1D; Supplementary Figure S1). Several lines, such as 1G-1 and 30A, displayed a more complex integration pattern (Figure 1B; Supplementary Figure S1).

We analyzed the accumulation of GNA in all lines by semi-quantitative immunoblot analysis of TSP from clarified extract of culms and leaves. The yield of GNA from the single-promoter *pUbi:GNA* lines varied in culms from low (< 0.1 mg kg^–1^ culm weight or < 0.01% TSP; 54.0% of 115 recovered plants), to moderate (≥ 0.1 mg kg^–1^ culm weight or ≥ 0.01% TSP; 22.7% of 115 recovered plants) and high (0.3 mg kg^–1^ culm weight or 0.04% TSP; 23.3% of 115 recovered plants) (Table 1). The GNA yield range was 0.07–0.30 mg kg^–1^ culm weight (0.01–0.04% TSP) (Table 1).

**TABLE 1:** Recombinant GNA yield in transgenic sugarcane and energy cane culms.

	**GNA yield as determined by semi-**	
	**quantitative immunoblot analysis**	
***GNA*-expressing line and percentage of plants analyzed**	**GNA (mg kg^–1^ culm weight)**	**TSP (%)**
**Sugarcane**		

**Single-promoter:*GNA* lines**		
*pU:GNA* double-terminator (*35STNOST*) lines (27 lines; 115 plants)	0.07–0.3 (range)	0.01–0.04 (range)
54.0%	< 0.1	< 0.01
22.7%	≥ 0.1	≥ 0.01
23.3%	≥ 0.3	≥ 0.04
**Triple-promoter:*GNA* lines**		
*pUBD5-1:GNA* double-terminator (*35STNOST*) (11 lines; 49 plants)	0.34–12.7 (range)	0.05–1.8 (range)
51.0%	1.2–12.7	0.2–1.8
49.0%	0.34–1.1	0.05–0.1

**Energy cane**		

**Quadruple-promoter:*GNA* lines**		
*pUBED5-1*:*GNA* double-terminator (*35STNOST*) (3 lines; 9 plants)	0.9–8.3 (range)	0.12–1.2 (range)
40.0%	2.7–8.3	0.37–1.2
20.0%	1.6–1.7	0.22–0.24
40.0%	0.9–1.4	0.12–0.19

*The percentage (%) of plants carrying the single or stacked promoter:*GNA* is presented under each line category. *U*, maize *ubiquitin 1* promoter; *B*, promoter for *Sugarcane bacilliform virus*; *E*, promoter for sugarcane *elongation factor 1*α; *D5-1*, promoter for sugarcane *dirigent5-1*; *35ST*, *Cauliflower mosaic virus* 35S terminator; *NOST*, *Agrobacterium tumefaciens* nopaline synthase terminator; TSP, Total soluble protein.*

The yield of GNA from the single-promoter *pUbi:GNA* lines was higher in leaves than in culms (Tables 1, 2). The yield range of GNA was 1.60–3.82 mg/kg^–1^ of leaf material (0.1–0.3% TSP), with 70% of plants producing GNA in the range 1.60–2.48 mg kg^–1^ of tissue (0.10–0.19% TSP) and 30% in the range 2.60–3.82 mg/kg^–1^ of tissue (0.2–0.3% TSP) (Table 2). Therefore, a 7.5-10 fold increase in GNA yield was achieved in the leaves of the single-promoter *pUbi:GNA* lines.

**TABLE 2:** Recombinant GNA yield in transgenic sugarcane leaves.

	**GNA yield as determined by semi-**	
	**quantitative immunoblot analysis**	
***GNA*-expressing line and percentage of plants analyzed**	**GNA (mg kg^–1^ leaf weight)**	**TSP (%)**
**Single-promoter:*GNA* lines**		
*pU:GNA* double-terminator (*35STNOST*) lines (27 lines; 115 plants)	1.6–3.82 (range)	0.1–0.3 (range)
70.0%	1.6–2.48	0.1–0.19
30.0%	2.6–3.82	0.2–0.3
**Triple-promoter:*GNA*lines**		
*pUBD5-1:GNA* double-terminator (*35STNOST*) (11 lines; 49 plants)	3.96–29.3 (range)	0.31–2.3 (range)
85.0%	3.96–7.0	0.31–1.0
15.0%	7.7–29.3	1.1–2.3

*The percentage (%) of plants carrying the single or stacked promoter:*GNA* is presented under each line category. *U*, *maize ubiquitin 1* promoter; *B*, promoter for *Sugarcane bacilliform virus*; *E*, promoter for sugarcane *elongation factor 1*α; *D5-1*, promoter for sugarcane *dirigent5-1*; *35ST*, *Cauliflower mosaic virus* 35S terminator; *NOST*, *Agrobacterium tumefaciens* nopaline synthase terminator; TSP, Total soluble protein.*

### The Accumulation of Recombinant GNA in Transgenic Sugarcane and Energy Cane Is Enhanced by Promoter Stacking

**FIGURE 2:**
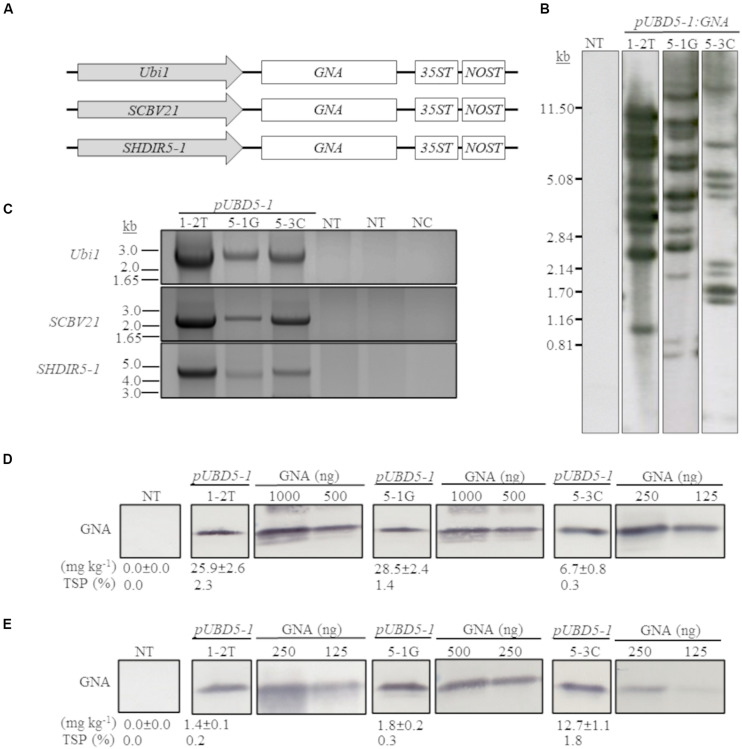
Molecular characterization of the stacked promoter:*GNA*-expressing lines. **(A)** Design of a representative stacked promoter:recombinant *GNA* expression system developed for sugarcane. Promoters used include the constitutive maize *ubiquitin 1* (*Ubi1*; *pU*) or the culm-regulated promoters from *Sugarcane bacilliform virus* (*SCBV21; B*) and sugarcane *dirigent5-1* (*SHDIR5-1*; *D5-1*). *35ST*: terminator derived from *Cauliflower mosaic virus* 35S RNA; *NOST*: *Agrobacterium tumefaciens* nopaline synthase terminator. Vector assembly and cloning sites are described under ‘MATERIALS AND METHODS.’ **(B)** Stable integration of recombinant *GNA* in three representative sugarcane triple-promoter *pUBD5-1:GNA* lines as detected by Southern blot analysis. DNA gel blots were hybridized to a probe corresponding to the *GNA* coding region. **(C)** Integration of the multiple expression cassettes for the triple promoter:*GNA* stacked lines was confirmed by polymerase chain reaction using primers specific to each promoter. Detection of *Ubi1* promoter with primer set *pUbi*-Fl/35ST-R (2.5 Kb fragment), detection of *SCBV21* promoter with primer set *pSCBV21*-F/35ST-R (2.179 Kb fragment) and detection of *pSHDIR5-1* with primer set *pSHDIR5-1*-F*/*GNA127-R (4.88 Kb fragment). GNA accumulation in sugarcane leaves **(D)** and culms **(E)** of three representative sugarcane *pUBD5-1:GNA* lines (shown as *pUBD5-1* in Figure) as assessed by quantitative immunoblot analysis of total soluble proteins (TSP), using a polyclonal anti-GNA antibody. Molecular weight of GNA is ∼12 kDa. All immunoblots were loaded with an equal amount of TSP (60 μg) per lane and 25, 62.5, 125, 250, 500 or 1000 ng of snowdrop-bulb native GNA as a standard. The GNA yield (mg kg^–1^ of tissue) in clarified juice extract is indicated. Yield values represent two biological replicates and are reported with the standard error. NT: non-transformed (tissue culture derived) plant; NC: no DNA template. The full-length uncropped DNA autoradiograms are displayed in Supplementary Figure S1, and the TSP immunoblots in Supplementary Figures S5, S6.

We previously developed and successfully used a combinatorial stacking gene-promoter expression system to significantly enhance the accumulation of recombinant bovine lysozyme (BvLz) in sugarcane culms (Damaj and Mirkov, 2019). The system consists of co-expressing the gene from multiple constitutive or culm-regulated promoters on separate expression vectors via combinatorial plant transformation. Culm tissue constitutes the largest fraction of harvestable biomass and represents a suitable tissue for the large-scale production of bulk proteins. To test whether the recombinant GNA levels recovered from the culms of the single-promoter *pUbi:GNA* lines was enhanced by using multiple promoter:*GNA* sequences, we co-transformed 2-month-old embryogenic calli of sugarcane (CP72-1210, TCP87-3388 and CP89-2143) and energy cane (CP10-4928 and Ho02-113) with the multiple promoter:*GNA* expression vectors (Figure 2A), using the *bar* gene (phosphinothricin acetyl transferase) as a selectable marker. In total, 11 independent transgenic *GNA* lines (49 plants) generated from the combinatorial transformation of sugarcane with the triple-promoter:*GNA* expression vectors were identified by Southern blot analysis (Figure 2B). The size of each respective *GNA* expression vector (promoter, *GNA* and double terminator) in the stacked triple-promoter:*GNA* lines was confirmed by PCR using primers encompassing each of the different promoter:*GNA*-terminator cassettes (Figure 2C and Supplementary Figure 3).

Most of the sugarcane triple-promoter:*GNA* lines exhibited a complex integration pattern (> 8 integration events) by Southern blotting (Figure 2B; representative *pUBD5-1:GNA* lines 1-2T, 5-1G and 5-3C; Supplementary Figure S1). No phenotypic differences were observed between the transgenic sugarcane *GNA* lines and non-transformed plants (Supplementary Figure S2), as previously observed with transgenic sugarcane *BvLz* plants (Damaj and Mirkov, 2019).

We analyzed the accumulation of GNA in all lines by semi-quantitative immunoblot analysis of TSP from clarified extract of culms and leaves. The GNA yield of transgenic sugarcane lines from the stacked triple-promoter *pUbi-pSCBV21-pSHDIR5-1*(*pUBD5-1*)*:GNA* increased in culms by up to about 42.3-fold (up to 12.7 mg kg^–1^ of tissue or 1.8% TSP) compared to the yield in single-promoter *pUbi:GNA* lines (pU:GNA; 0.3 mg kg^–1^ of tissue or 0.04% TSP), with 49% of plants containing 0.34–1.1 mg kg^–1^ tissue (0.05–0.100% TSP) and 51% containing 1.2–12.7 mg kg^–1^ of tissue (0.20–1.8% TSP) (Table 1).

The GNA yield of transgenic lines containing the stacked triple-promoter *pUBD5-1:GNA* was also enhanced in leaves, by about 7.7-fold (up to 29.3 mg kg^–1^ of tissue or 2.3% TSP) more than that of the single-promoter *pUbi:GNA* lines, with 85% of plants containing 3.96–7.00 mg GNA kg^–1^ of tissue (0.31–1.00% of TSP) and 15% containing between 7.7–29.3 mg GNA kg^–1^ of tissue (1.1–2.3% of TSP) (Table 2).

We also generated transgenic sugarcane lines containing stacked quadruple-promoter:*GNA* expression vectors, but the maximum yield of GNA obtained was 9.4 mg kg^–1^ of tissue (1.3% TSP). Therefore, stacking more than three promoter:*GNA* vectors did not further enhance GNA yield. Furthermore, the yield of GNA from energy cane transformed with *GNA* driven from four stacked promoters (*pUbi-pSHEF1*α*-pSCVB21-pSHDIR5-1:GNA*) was comparable to that obtained from quadruple-stacked promoter:*GNA* sugarcane. Three lines (nine plants) were generated that accumulated GNA to levels ranging from low at 0.9–1.4 mg kg^–1^ (0.10–0.19% TSP) (40% of plants), to moderate at 1.6–1.7 mg kg^–1^ (0.22–0.24% TSP) (20% of plants), and high at 2.7–8.3 mg kg^–1^ of culm (0.37–1.20% TSP) (40% of plants) (Table 1).

A comparison of the GNA yield in the sugarcane single-promoter *pUbi:GNA* line 1G-1 (3.6 mg kg^–1^ of leaf and 0.20 mg kg^–1^ of culm) (Figures 1C,D; Supplementary Figure S4) with that of the triple-promoter *pUBD5-1:GNA* line 1-2T (25.9 mg kg^–1^ leaf tissue and 1.4 mg kg^–1^ culm tissue) (Figures 2D,E; Supplementary Figures S5, S6), which contained about 14 *GNA* insertions (Figures 1B, 2B; Supplementary Figure S1), showed that there was a clear increase in GNA yield irrespective of the number of transgenic insertions. This suggests that the increase in GNA yield is primarily correlated with the number of combinatorial stacked promoters and is not dependent on the number of *GNA* insertions alone, which was reported for combinatorial promoter:*BvLz* stacking in sugarcane (Damaj and Mirkov, 2019).

Our results show that levels of GNA can be enhanced by stacking or co-expressing the gene under the control of multiple different promoters. We speculate that the observed additive increase is mainly due to increased transcriptional output from the different transgene units stably integrated into the plant genomes. However, often multiple copies do not result in greater expression. It has been noted that integration of multiple transgenes, often in tandem, using same promoter can lead to homology-dependent gene silencing (HDGS) via DNA methylation events (Matzke et al., 1989, 1994; Tu et al., 2000; Bock, 2013; Rajeevkumar et al., 2015). This can lower the net expression (Assaad et al., 1993; Kumpatla and Hall, 1998; Pawlowski and Somers, 1998; Kanno et al., 2000; Khaitová et al., 2011). In our system, HDGS may not be an issue mainly due to the use of different promoter sequences and separate vectors that would avoid tandem integration of transgenes.

Thus, increasing the expression level of stably transformed recombinant *GNA* sequences through combinatorial promoter-gene stacking proved to be an efficient approach to increase protein accumulation and resulted in high GNA levels that typically approached 1.8% and 2.3% of the TSP from transgenic culms (12.7 mg kg^–1^ of tissue) (Table 1) and leaves (29.3 mg kg^–1^ of tissue) (Table 2), respectively.

### The Accumulation of Recombinant GNA Is Responsive to Stress-Regulated Hormones

**FIGURE 3:**
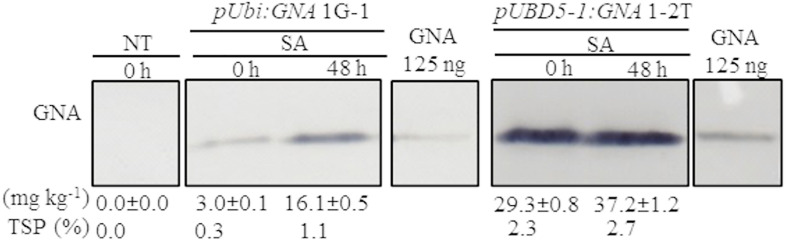
Accumulation of recombinant GNA is enhanced by the stress-signaling hormone salicylic acid (SA) in single and triple-promoter:*GNA*-expressing sugarcane lines. GNA accumulation was determined by quantitative immunoblot analysis in total soluble protein (TSP) extracted from leaf tissue of representative *pUbi:GNA* and *pUBD5-1:GNA* lines at 0 and 48 h of SA treatment (5 mM), using a polyclonal anti-GNA antibody. Molecular weight of GNA is ∼12 kDa. All immunoblots were loaded with an equal amount of TSP (60 μg) per lane and 125 ng of snowdrop-bulb native GNA as a standard. The GNA yield (mg kg^–1^ of tissue) in clarified juice extract is indicated. Yield values represent two biological replicates and are reported with the standard error. Molecular weight of GNA is ∼12 kDa. *pUbi* and *pU*: maize *ubiquitin 1* promoter; *B*: promoter for *Sugarcane bacilliform virus; D5-1*: promoter for sugarcane *dirigent5-1*. NT: non-transformed (tissue culture derived) plant. The full-length uncropped TSP immunoblots are displayed in Supplementary Figure S7.

Another approach to increase the level of recombinant GNA is to induce promoter activity in transgenic plants by applying stress-regulated hormones. The *Ubi* and *SHDIR16* promoters can be induced by stress-signaling hormones such as salicylic acid (SA) (Damaj et al., 2010). To test whether SA can further induce *GNA* expression, we sprayed and irrigated several representative single-promoter *pUbi:GNA* and triple-promoter *pUBD5-1:GNA* sugarcane lines with SA for 48 h in the greenhouse. The level of GNA accumulation in leaves increased by 5.4-fold (from 3.0 to 16.1 mg kg^–1^ of tissue) following SA application in the single-promoter *pUbi:GNA* 1G-1 line and by 1.3-fold (from 29.3 to 37.2 mg kg^–1^ of tissue) in the triple-promoter *pUBD5-1:GNA* line 1-2T (Figure 3; Supplementary Figure S7). These results corroborate those of previous studies (Damaj and Mirkov, 2019), and the patterns of SA induction of recombinant GNA are consistent with the kinetics of promoter activation by SA (Damaj et al., 2010). The use of promoters such as *pUbi*, *pSHDIR*s and *pSCBV21* that can be turned on and off by stress-regulated hormones such as SA holds great potential for the bioengineering industry, as a means to increase the production of GNA and other recombinant therapeutic proteins.

### Optimization of the Bench-Scale Extraction of Recombinant GNA From Transgenic Sugarcane and Energy Cane

**FIGURE 4:**
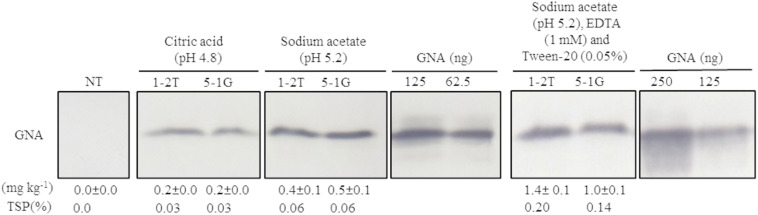
Optimization of recombinant GNA extraction in sugarcane transgenic culms. Accumulation of recombinant GNA was assessed by semi-quantitative immunoblot analysis of representative lines expressing *GNA* from the three promoters, maize *ubiquitin 1*, *Sugarcane bacilliform virus* and sugarcane *dirigent5-1*, using a polyclonal anti-GNA antibody. All immunoblots were loaded with an equal amount of TSP (60 μg) per lane and 62.5, 125 or 250 ng of snowdrop-bulb native GNA as a standard. Molecular weight of GNA is ∼12 kDa. The GNA yield (mg kg^–1^ of tissue) in clarified juice extract is indicated. Yield values represent two biological replicates and are reported with the standard error. NT: non-transformed (tissue culture derived) plant. The full-length uncropped TSP immunoblots are displayed in Supplementary Figure S9.

Because no previous attempts have been made to extract GNA from sugarcane culms, we first evaluated a bench-scale extraction of recombinant GNA from transgenic culms of sugarcane and energy cane. We tested two different buffers to extract recombinant GNA from the culms of sugarcane and energy cane that expressed the triple-promoter *pUBD5-1:GNA*, with *GNA* expression driven by *pUbi* (U), *pSCBV21* (B) and *pSHDIR5-1* (D5-1), sodium acetate buffer (pH 5.2) and citric acid (pH 4.0), as outlined in Supplementary Figure S8 (described in section “MATERIALS AND METHODS”). The GNA yield of representative *pUbi-pSCBV21-pSHDIR5-1(pUBD5-1):GNA* lines 1-2T and 5-1G was 0.4 and 0.5 mg kg^–1^, respectively (0.06% TSP for both), using sodium acetate (pH 5.2), and 0.2 and 0.2 mg kg^–1^, respectively (0.03% of TSP for both lines), with citric acid (Figure 4; Supplementary Figure S9); this represented a 2.0- to 2.5-fold increase in GNA yield with sodium acetate compared to that with citric acid.

We then further optimized the extraction of recombinant GNA from culm tissue by using sodium acetate buffer (pH 5.2) supplemented with 0.05 mM Tween 20 (v/v) (a mild surfactant) and 1 mM EDTA. This increased the yield of GNA from 0.4 mg kg^–1^ (0.06% TSP) to 1.4 mg kg^–1^ (0.2% TSP) for *pUBD5-1*:*GNA* line 1-2T and from 0.5 mg kg^–1^ (0.06% TSP) to 1.0 mg kg^–1^ (0.14% TSP) for *pUBD5-1*:*GNA* line 5-1G (Figure 4; Supplementary Figure S9). This represented at least a 2.0- to 3.5-fold increase following the addition of Tween 20 and EDTA. Surfactants are commonly added to protein extraction buffers to increase cell wall and membrane breakage and thus potentially increase the amount of extracted protein. To our knowledge, this is the first attempt to extract recombinant GNA from culms and to recover high amounts of protein.

We also optimized the extraction of recombinant GNA from transgenic sugarcane leaves. Under our experimental conditions, Tris–HCl (pH 6.8), which is a common buffer for the extraction of GNA from leaves, did not yield detectable GNA (data not shown). However, using an optimized 0.2 M sodium acetate/0.2 M acetic acid buffer (pH 5.2), we could recover GNA from transgenic sugarcane leaves at a level up to 29.3 mg kg^–1^ of tissue (2.3% TSP). This yield is higher than that previously reported for sugarcane and other monocot crops. Accumulation levels of recombinant GNA of 0.01–0.25% and 0.13–0.28% TSP have been obtained from rice leaves using Tris–HCl (pH 9.0) (Rao et al., 1998) and from maize leaves using phosphate buffer (with 1% [v/v] β-mercaptoethanol and 10% [v/v] glycerol). A higher recombinant GNA yield of 0.89% TSP has been achieved from lyophilized sugarcane leaves using Tris–HCl (pH 6.8) with 2% (w/v) SDS, 10% (v/v) glycerol (Wang Z. et al., 2005) and 5% (v/v) β-mercaptoethanol as an extraction buffer (Setamou et al., 2002).

### Recombinant GNA Accumulates Differentially in Transgenic Culms and Leaves

In the expression system here, recombinant GNA differentially accumulated in the leaves and culms of transgenic sugarcane plants. For example, the GNA yield from the representative single-promoter *pUbi:GNA* lines 1D, 1G-1 and 30A was 6.0- 18-, and 14.5-fold higher in leaves (0.60, 3.6 and 2.9 mg kg^–1^ of tissue) than in culms (0.10, 0.20 and 0.2 mg kg^–1^ of tissue) (Figures 1C,D; Supplementary Figure S4).

The GNA yield from representative sugarcane triple-promoter *pUBD5-1:GNA* lines 1-2T, 5-1G and 5-3C was 2.3, 1.4 and 0.30% TSP (25.9, 28.5 and 6.7 mg kg^–1^, respectively) in leaf tissue (Figure 2D; Supplementary Figure S5), and 0.20, 0.3 and 1.8% TSP (1.4, 1.8 and 12.7 mg kg^–1^, respectively) in culm tissue (Figure 2E; Supplementary Figure S6). This represented an 18.5- and 15.8-fold higher GNA accumulation for lines 1-2T and 5-1G, respectively, in leaf than in culm tissue, and 1.9-fold more GNA for line 5-3C in culms than in leaves (Figures 2D,E; Supplementary Figures S5, S6). These results suggest that the accumulation of GNA is differentially regulated between sugarcane leaves and culms. We speculate the differences could be attributed to the contrasting biochemical compositions of the two tissue types. Sugarcane culms are mainly composed of soluble sugars and lignocellulosic fiber, with low levels of native protein (Palaniswamy et al., 2016). While leaves have lower fiber content and proportionally more biochemically active cells, which could lead to greater transcriptional output. One could technically combine either tissue types (leaves or culms) for GNA extraction, and as such are not mutually exclusive. However, extraction of proteins from culm tissue may be practical since most of the harvesting and processing equipment used for sugarcane and extracting sugar can be readily extended for purification of recombinant proteins from culms.

### Purification and MALDI-TOF Characterization of Sugarcane Recombinant GNA

**FIGURE 5:**
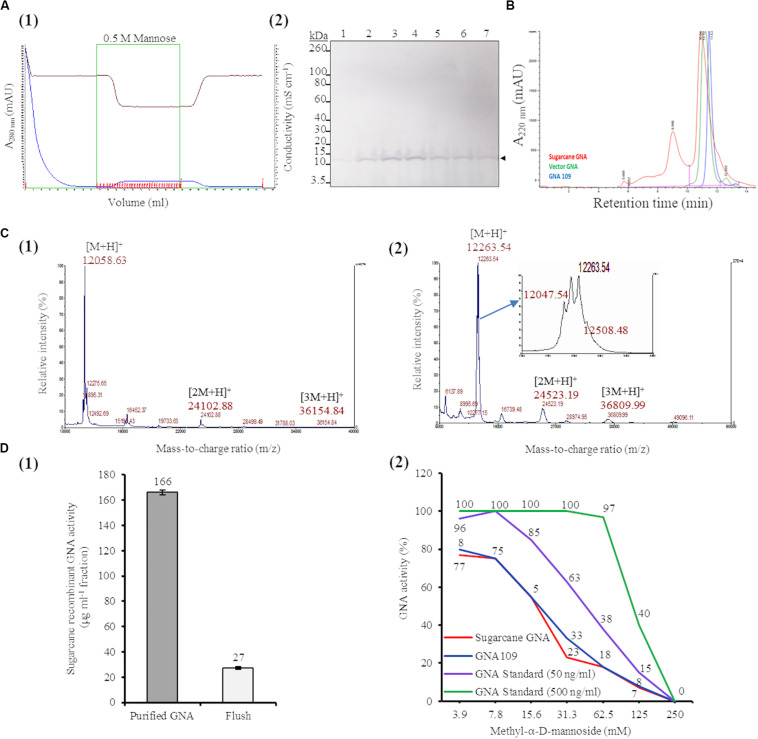
Characterization of sugarcane recombinant GNA. **(A)** Purification of GNA from transgenic sugarcane leaves by mannose-agarose affinity chromatography. (1) Chromatogram of eluted mannose-bound fractions. Blue line: chromatogram of UV absorbance at 280 nm (A_280__nm_; mAU) to monitor eluted fractions; brown line: chromatogram of conductivity (mS cm^–1^), which reflects the ionic strength of the buffer; green line: chromatogram of concentrated fractions. Eluted fractions are marked in red. (2) Immunoblot analysis of eluted fractions, using a polyclonal anti-GNA antibody. Lanes 1 to 7 represent elution fractions 1.A.7, 1.A.8, 1.A.9, 1.A.10., 1.A.11, 1.A.12 and 1.B.1, respectively, as shown in the chromatogram. The GNA protein band is indicated by an arrow. The full-length uncropped immunoblot is displayed in Supplementary Figure S10. **(B)** Analysis of purified sugarcane recombinant GNA under non-reduced conditions by size-exclusion chromatography at absorbance of 220 nm over time. The chromatogram of sugarcane recombinant GNA is overlaid with those of GNA_109_ and the snowdrop-bulb native GNA standard (Vector GNA). **(C)** Analysis of purified sugarcane recombinant GNA under non-reduced conditions using Matrix-Assisted Laser Desorption Ionization Time-of-Flight Mass Spectrometry (MALDI-TOF MS). (1) The MS spectra of sugarcane GNA displaying molecular weights of *m/z* 12,058.63 [M + H]^+^, *m/z* 24,102.88 [2M + H]^+^ and *m/z* 36,154.84 [3M + H]^+^, which represent monomer, dimer and trimer sugarcane GNA, respectively. (2) The MS spectrum of snowdrop-bulb native GNA standard displaying three additional peaks along with actual *m/z* of 12,048.63 [M + H]^+^ (monomer) (enlarged spectrum area). **(D)** Competitive binding assay of recombinant GNA with mannan-binding inhibitor, methyl-α-D-mannoside. (1) Estimation of activity of purified sugarcane recombinant GNA with enzyme-linked lectin assay. Sugarcane recombinant GNA activity is expressed in μg mL^–1^ of pooled pure enriched GNA fractions from Figure 5A. (2) GraphPad prism analysis of inhibition assay with purified sugarcane recombinant GNA, GNA_109_ and GNA standard (500 ng mL^–1^ and 50 ng mL^–1^), using two-fold serial dilutions of the inhibitor (3.9, 7.8, 15.6, 31.3, 62.5, 125 and 250 mM). GraphPad prism data conversion of absorbance at 450 nm into percentage of activity is recorded.

We purified sugarcane recombinant GNA from TSP extracts from a pool of leaves (0.89 kg of tissue; GNA yield of 7.71 mg kg^–1^ tissue weight) from seven representative single- and triple-promoter *GNA* lines in parallel with recombinant GNA_109_ and the snowdrop-bulb native GNA (Vector Labs) using immobilized mannose. Following enrichment of mannose-binding proteins in the sugarcane recombinant GNA sample by mannose-agarose affinity chromatography (Figure 5A1), we detected a polypeptide band corresponding to mature GNA (about 12.0 kDa) by immunoblot analysis of SDS-PAGE of mannose-bound elution fractions (Figure 5A2; Supplementary Figure S10). We performed analytical characterization of GNA after concentration of the enriched elution GNA fractions using a 5.0-kDa-MWCO tangential flow filtration device (about 20-fold). The analysis of purified sugarcane recombinant GNA by size-exclusion (SE) chromatography under non-reducing conditions showed one major peak at 10.89 min and a small peak at 8.9 min (Figure 5B). The purities of the recombinant GNA monomer and tetramer were 65.5% and 24%, respectively, at 8.9 min. We compared the sugarcane GNA SEC chromatogram with that of the snowdrop-bulb native GNA (*G. nivalis* agglutinin; 1NIV, Protein Data Bank accession number 1NIV_A) and recombinant GNA_109_ (*G. nivalis* lectin, LECGNA2 with four additional amino acids, Thr_106_ His_107_ Thr_108_ Gly_109_, at its C-terminus; high mannose-binding activity; (Raemaekers, 2000). Snowdrop-bulb GNA and GNA_109_ displayed a major peak at 11.12 and 11.43 min, respectively (Figure 5B), close to the major peak observed for sugarcane recombinant GNA.

To determine the molecular weight of sugarcane recombinant GNA protein, we performed MALDI-TOF MS analysis under non-reducing conditions. The MS spectra of sugarcane recombinant GNA displayed one peak with a mass-to-charge ratio (*m*/*z*) of 12,058.63 [M + H]^+^, representing the GNA monomer (Figure 5C1). We also detected peaks of *m*/*z* 24,102.88 [2M + H]^+^ and *m*/*z* 36,154.84 [3M + H]^+^, which represented the GNA dimer and trimer, respectively (Figure 5C1). For comparison, the recombinant GNA_109_ MS spectrum also showed a single peak, similar to sugarcane recombinant GNA, with an *m*/*z* of 12,052.08 [M + H]^+^ representing the GNA_109_ monomer (Supplementary Figure S11). The theoretical amino-acid sequence molecular weight for GNA_109_ is 12,051.42 Da. The snowdrop-bulb native GNA (Vector Labs) spectrum revealed three additional peaks (12,263.54 and 12,508.48), as well as the peak corresponding to the actual *m*/*z* of 12,047.54 [M + H]^+^ (monomer) (Figure 5C2, enlarged peak area). The theoretical molecular weight of snowdrop-bulb native GNA is 12,054.41 Da. These results demonstrate that sugarcane recombinant GNA has very similar molecular weight MS peak characteristics and functionality in comparison to the native snowdrop-bulb GNA protein.

### Recombinant GNA Retains Binding Specificity to Mannan

The binding efficiency of purified sugarcane recombinant GNA protein to immobilized mannan was determined by ELLA. This assay allows specific lectin-mannose interactions to be analyzed and forms the basis for a functional assay that quantitatively estimates the amount of GNA (Gornik and Lauc, 2007; Couzens et al., 2014). Sugarcane recombinant GNA in the purified sample bound to mannan with an estimated specific activity of 166 μg mL^–1^ based on a standard curve with snowdrop-bulb native GNA (Figure 5D1).

Most lectins synthesized in plants were initially characterized using inhibition assays, in which monosaccharides or their derivatives were used to block lectin binding sites; the lectins were then grouped by their specificity for the monosaccharide(s) that inhibited their binding at millimolar concentrations (Cummings et al., 2017). Therefore, we confirmed the specificity of the interaction between recombinant sugarcane GNA and mannan through an inhibition assay with methyl-α-D-mannoside, a competitive inhibitor of mannan binding (Raemaekers, 2000; Sommer et al., 2018). Equal concentrations of sugarcane recombinant GNA, GNA_109_ and snowdrop-bulb native GNA were incubated with two-fold serial dilutions (3.9, 7.8, 15.6, 31.3, 62.5, 125.0 and 250.0 mM) of methyl-α-D-mannoside (Figure 5D2). The inhibition assay revealed that the mannan-binding activity of all three GNAs decreased with an increase in mannoside concentration, as expected, with binding being reduced to a minimum at a limiting concentration of 250 mM mannoside. Sugarcane recombinant GNA and GNA_109_ showed a similar rapid reduction in binding activity with an increase in the inhibitor, compared to the delayed reduction in the level of the snowdrop-bulb native GNA (Figure 5D2). At a low (3.9 mM) inhibitor concentration, the activities of all three GNAs were only slightly affected; the sugarcane recombinant GNA and GNA_109_ activities decreased by about 20% (from 100% to 80%) compared to that of the snowdrop-bulb native GNA, which remained at 100%. However, the inhibitor concentration required to reduce mannan-binding activity to about 55% was much lower for sugarcane recombinant GNA and GNA_109_ (15.6 mM of inhibitor) than for the native GNA standard at 50 ng mL^–1^ (31.3 mM inhibitor) (Figure 5D2). The mannan-binding activity for all three GNAs decreased to 0% at an inhibitor concentration of 250 mM.

The ELLA and mannan inhibition assay indicated that sugarcane recombinant GNA, GNA_109_ and snowdrop-bulb native GNA were all able to selectively bind to the anti-GNA antibody and the mannan substrate. However, sugarcane recombinant GNA and GNA_109_ possessed similar mannan-binding activities and were more sensitive to mannan inhibition than snowdrop-bulb native GNA.

To check whether any sequence differences existed that might affect the binding selectivity and affinity of the sugarcane recombinant GNA to the anti-GNA antibody or the mannan substrate, we compared the translated peptide sequence used to transform sugarcane (*G. nivalis* lectin; LECGNA2, Protein Bank accession number AAA33346) with that of the mature snowdrop-bulb native GNA (*G. nivalis* agglutinin; 1NIV, Protein Data Bank accession number 1NIV_A). The amino-acid alignment of the two proteins (Supplementary Figure S12) showed that the sequences of the mature GNA protein region were identical in both GNA proteins, with LECGNA2 N and C terminal signal sequences that are presumably cleaved in sugarcane and snowdrop. Both GNA proteins contain the consensus sequence motif QXDXNXVXY (QEDCNLVLY) (Supplementary Figure S12), which is involved in recognition of the α-D-mannose substrate.

The MALDI-TOF MS data showed that the spectrum of the snowdrop-bulb native GNA displayed three additional peaks in addition to that of the actual molecular weight of *m*/*z* 12,047.54 [M + H]^+^ (Figure 5C2), compared to the spectra of sugarcane recombinant GNA and GNA_109_. This suggests that the snowdrop-bulb native GNA is composed of more than one isoform. This is consistent with reports about the presence of multiple isoforms or isolectins in the snowdrop-bulb native GNA (Van Damme et al., 1991), which reflect sequence variability in the C-terminal amino-acid region. Although these isolectins have a similar overall specificity and share a high sequence similarity, it is unclear whether they possess identical biological activities (Peumans and Van Damme, 1995). This is an important consideration when expressing these isolectins in transgenic plants, and individual lectins should be tested to identify individual activity. In our study, only one GNA isolectin was synthesized and characterized in sugarcane. Further assessment of the qualities of the other native snowdrop-bulb GNA isoforms would be useful to select the most potent one and compare it with sugarcane recombinant GNA.

## Conclusion

Sugarcane and energy cane represent promising candidates as biofactories for the production of recombinant therapeutic snowdrop GNA. In this work, a gene-promoter stacking approach resulted in levels of GNA that were 1.8% and 2.3% of TSP in transgenic culms and leaves, respectively. Our approach consisted of stacking multiple promoters to co-express recombinant *GNA* from separate expression vectors, using combinatorial transformation. High TSP levels of 2.7% were also achieved by inducing promoter activity in GNA transgenic lines with stress-regulated hormones such as SA. Sugarcane recombinant GNA purified by mannose-agarose affinity chromatography and native snowdrop-bulb GNA exhibited a similar binding specificity to the anti-GNA antibody and mannan substrate. Our results suggest that high levels of GNA (up to 2.7% TSP) can be successfully isolated from transgenic sugarcane and energy cane tissues.

## Data Availability Statement

All datasets presented in this study are included in the article/Supplementary Material.

## Author Contributions

CP, MD, Z-NY, JM, JD, and KM designed the experiments. MD, CP, and Z-NY generated the GNA constructs. CP, MD, NS-G, and JM conducted plant transformation. CP, MD, and JM screened transgenic plants. CP and MD performed hormone experiments. CP, MD, JM, and NS-G conducted small-scale protein extraction and western blot analyses. BB and EW performed the large-scale purification and characterization of the sugarcane recombinant protein. CP and MD prepared the manuscript. JD and KM supervised the study and reviewed the manuscript. All authors contributed to the article and approved the submitted version.

## Conflict of Interest

BB and EW were employed by the companies iBio (Bryan, TX) and MDx BioAnalytical Laboratory (College Station, TX) respectively. The remaining authors declare that the research was conducted in the absence of any commercial or financial relationships that could be construed as a potential conflict of interest.

## Supplementary Material

FIGURE S1 **(A,B)** Full-length uncropped DNA gel blot autoradiograms used to prepare Southern blots in Figures 1B, 2B, respectively. **(A)** Single-promoter *pUbi:GNA* lines 1G-1: lane 8, 1D: lane 5, and 30A: lane 18. **(B)** Triple-promoter *pUBD5-1:GNA* lines 1-2T: lane 3, 5-1G: lane 7, and 5-3C: lane 4. Non-transformed control plant: lane 1 in both blots. **(A,B)** Full-length uncropped DNA gel blot autoradiograms used to prepare Southern blots in Figures 1B, 2B, respectively.

FIGURE S2 Qualitative phenotype of the triple-promoter:*GNA*-expressing sugarcane lines. One-year-old mature plants are shown. GNA, *GNA* transgenic plants; WT, wild type plants.

FIGURE S3 **(A–C)** Uncropped agarose gel used to prepare Figure 2C. **(A)** Integration of *pUbi:GNA* expression cassettes: lane 1, 1 kb DNA plus ladder; lane 2, *pUBD5-1:GNA* line 1-2T; lane 3, *pUBD5-1:GNA* line 5-1G; lane 4, *pUBD5-1:GNA* line 5-3C; lanes 5 and 6: Non-transformed (NT) CP72-1210 and CP89-2143, respectively; lane 7, NC-no DNA template and lane 8, *pUbi:GNA* plasmid. **(B)** Integration of *SCBV21:GNA* expression cassettes: lane 1, 1 kb DNA plus ladder; lane 2, *pUBD5-1:GNA* line 1-2T; lane 3 *pUBD5-1:GNA* line 5-1G; lane 4, *pUBD5-1:GNA* line 5-3C; lanes 5 and 6: NT CP72-1210 and CP89-2143, respectively; lane 7, NC-no DNA template and lane 8, *pSCBV21:GNA* plasmid. **(C)** Integration of *pSHDIR5-1:GNA* expression cassettes: lane 1, 1 kb DNA plus ladder; lane 2, *pUBD5-1:GNA* line 1-2T; lane 3 *pUBD5-1:GNA* line 5-1G; lane 4, *pUBD5-1:GNA* line 5-3C (full and truncated amplicons); lanes 5 and 6: NT CP72-1210 and CP89-2143, respectively; lane 7, NC-no DNA template; and lane 8, *pSHDIR5-1:GNA* plasmid.

FIGURE S4 **(A,B)** Full-length uncropped immunoblot membranes used to prepare Figures 1C,D. **(A)** Uncropped immunoblot used to prepare Figure 1C (leaves) single-promoter *pUbi:GNA* lines 1G-1: lane 3 and 30A: lane 5 and GNA standard 125 ng: lane 13. **(B)** Uncropped immunoblot used to prepare Figure 1D (culms) single-promoter *pUbi:GNA* lines 1G-1: lane 3, 1D: lane 4 and 30A: lane 5 and non-transformed (NT): lane 11.

FIGURE S5 **(A–C)** Full-length uncropped immunoblot membranes used to prepare Figure 2D (Immunoblot for leaf samples). **(A)** GNA standard 1000 ng and 500 ng: lanes 2 and 3, respectively, and triple-promoter *pUBD5-1:GNA* line 1-2T: lane 10. **(B)** GNA standard 1000 ng and 500 ng: lanes 2 and 3, respectively, and triple-promoter *pUBD5-1:GNA* line 5-1G: lane 10. **(C)** GNA standard 250 ng and 125 ng: lanes 4 and 5, respectively, and *pUBD5-1:GNA* line 5-3C: lane 11.

FIGURE S6 **(A–C)** Full-length uncropped immunoblot membranes used to prepare Figure 2E (Immunoblot for culm samples). **(A)** GNA standard 250 ng and 125 ng: lanes 4 and 5, respectively, and triple-promoter *pUBD5-1:GNA* line 1-2T: lane 7. **(B)** GNA standard 500 ng and 250 ng: lanes 2 and 3; respectively, triple-promoter *pUBD5-1:GNA* line 5-1G: lane 10. **(C)** GNA standard 250 ng and 125 ng: lanes 2 and 3; respectively, and triple-promoter *pUBD5-1:GNA* line 5-3C: lane 10.

FIGURE S7 Full-length uncropped immunoblot membrane used to prepare Figure 3. Lanes 2 and 3 show single-promoter *pUbi:GNA* line 1G-1 before and after salicylic acid treatment, respectively; lanes 10 and 12 show triple-promoter *pUBD5-1:GNA* line 1-2T before and after salicylic acid treatment, respectively; lane 13 shows 125 ng of GNA standard.

FIGURE S8 Flowchart of the bench-scale extraction of recombinant GNA from transgenic sugarcane and energy cane culms.

FIGURE S9 **(A,B)** Full-length uncropped immunoblot membrane used to prepare Figure 4. **(A)** The following: GNA standard 125 ng and 62.5 ng: lanes 5 and 6, respectively; triple-promoter *pUBD5-1:GNA* lines 1-2T and 5-1G total soluble protein (TSP) extraction with citric acid pH 4.8: lane 8 and 9, respectively; TSP extraction of the same lines with sodium acetate pH 5.2: lanes 12 and 13, respectively. **(B)** GNA standard 250 ng and 125 ng: lanes 4 and 5, respectively, and triple promoter *pUBD5-1:GNA* lines 1-2T and 5-1G TSP extractions with sodium acetate (pH5.2)/EDTA/Tween-20: lanes 7 and 8.

FIGURE S10 Full-length uncropped immunoblot membrane used to prepare Figure 5A2. Lanes 1 to 7 represent elution fractions 1.A.7, 1.A.8, 1.A.9, 1.A.10., 1.A.11, 1.A.12, and 1.B.1, respectively.

FIGURE S11 Analysis of recombinant GNA_109_ under non-reduced conditions using Matrix-Assisted Laser Desorption Ionization Mass Spectrometry (MALDI-TOF MS). The MS spectra of GNA_109_ display the molecular weights of *m/z* 12,052.08 [M + H]^+^, *m/z* 24,103.60 [2M + H]^+^ and *m/z* 36,159.76 [3M + H]^+^, representing the monomer, dimer and trimer of GNA_109_, respectively.

FIGURE S12 Multiple alignment of amino-acid sequences of the translated GNA peptide transformed into sugarcane (*Galanthus nivalis* lectin; LECGNA2, Protein Bank accession number AAA33346) and the mature snowdrop-bulb native GNA (Vector Labs) (*Galanthus nivalis* agglutinin; 1NIV, Protein Data Bank accession number 1NIV_A). The consensus sequence motif QXDXNXVXY (QEDCNLVLY), involved in recognition of the α-D-mannose substrate, is boxed in red. Multiple amino-acid sequence alignment was performed using ClustalW 2.0 program (www.ebi.ac.uk/clustalw).

TABLE S1 Primers used for determination of promoter:*GNA* cassette integration in staked triple promoter:*GNA* sugarcane lines.
