# Supplementary material for: High-Level Production of Recombinant Snowdrop Lectin in Sugarcane and Energy Cane
Source: Front Bioeng Biotechnol. 2020 Aug 18;8:977. doi: 10.3389/fbioe.2020.00977 (PMC7461980; doi:10.3389/fbioe.2020.00977)
Supplement: FIGURE S1 — (A,B) Full-length uncropped DNA gel blot autoradiograms used to prepare Southern blots in Figures 1B, 2B, respectively. (A) Single-promoter pUbi:GNA lines 1G-1: lane 8, 1D: lane 5, and 30A: lane 18. (B) Triple-promoter pUBD5-1:GNA lines 1-2T: lane 3, 5-1G: lane 7, and 5-3C: lane 4. Non-transformed control plant: lane 1 in both blots. (A,B) Full-length uncropped DNA gel blot autoradiograms used to prepare Southern blots in Figures 1B, 2B, respectively. [file Data_Sheet_1.pdf]

Supplementary Material

High-Level Production of Recombinant Snowdrop Lectin in Sugarcane and Energy cane

Carmen S. Padilla[1¶], Mona B. Damaj[1¶], Zhong-Nan Yang[2], Joe Molina[1], Brian R. Berquist[3§], Earl L. White[4§], Nora Solís-Gracia[1], Jorge Da Silva[1,5] and Kranthi K. Mandadi[1,6*]

[1]*Texas A&M AgriLife Research and Extension Center, Weslaco, Texas 78596-8344, USA*
[2]*Institute for Plant Gene Function, Department of Biology, Shanghai Normal University, Shanghai, China*
[3]*iBio, 8800 HSC Parkway, Bryan, Texas 77808, USA*
[4]*MDx BioAnalytical Laboratory, Inc., 5890 Imperial Loop, Suite 12, College Station, Texas 77845, USA*
[5]*Department of Soil & Crop Sciences, Texas A&M University, College Station, Texas 77843-2474, USA*
[6]*Department of Plant Pathology & Microbiology, Texas A&M University, College Station, Texas 77843-2132, USA*

***Corresponding author:** Kranthi K. Mandadi, kkmandadi@tamu.edu

[¶§]Equal contributions

Running title: Recombinant therapeutic snowdrop lectin from *Saccharum* spp.

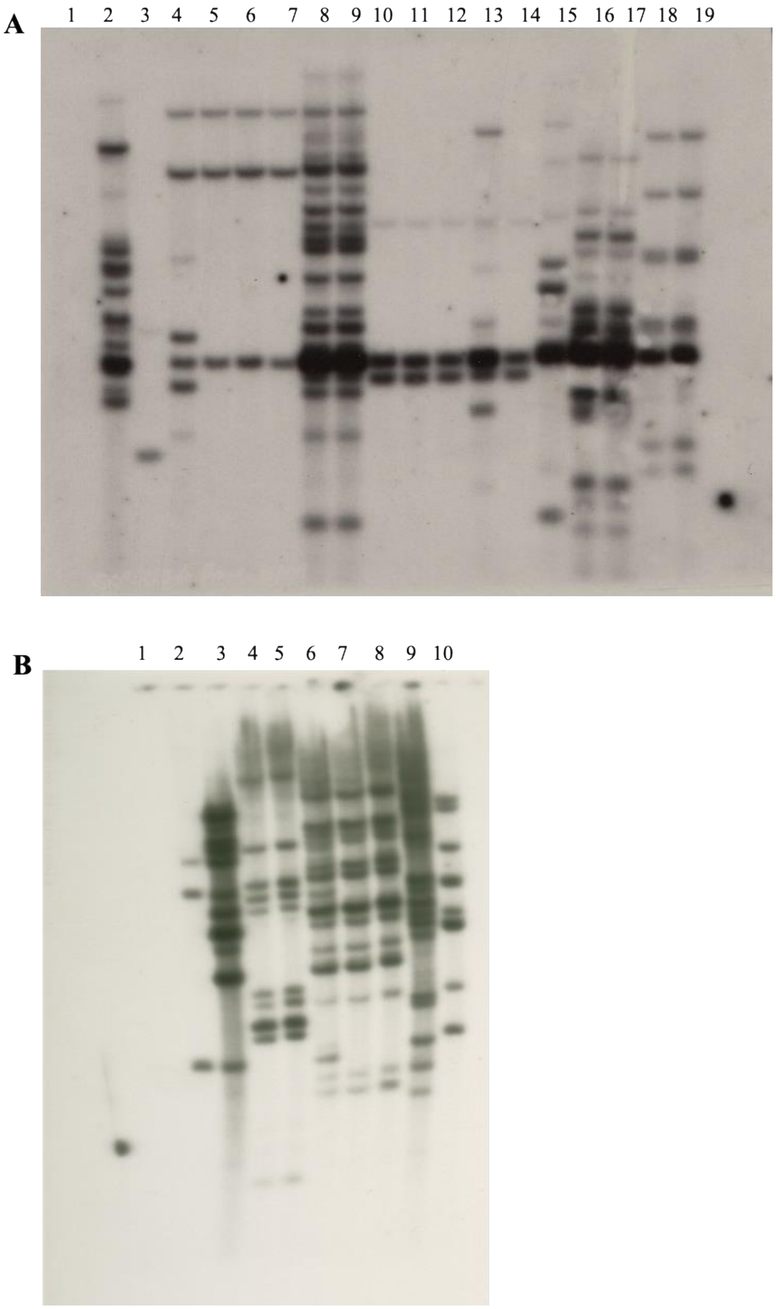

Supplementary Figure S1. (A) and (**B**) Full-length uncropped DNA gel blot autoradiograms used to prepare Southern blots in FIGURE 1B and FIGURE 2B, respectively. **(A)** Single-promoter *pUbi:GNA* lines 1G-1: lane 8, 1D: lane 5 and 30A: lane 18. **(B)** Triple-promoter *pUBD5-1:GNA* lines 1-2T: lane 3, 5-1G: lane 7 and 5-3C: lane 4. Non-transformed control plant: lane 1 in both blots. (**A**) and (**B**) Full-length uncropped DNA gel blot autoradiograms used to prepare Southern blots in FIGURE 1B and FIGURE 2B, respectively.

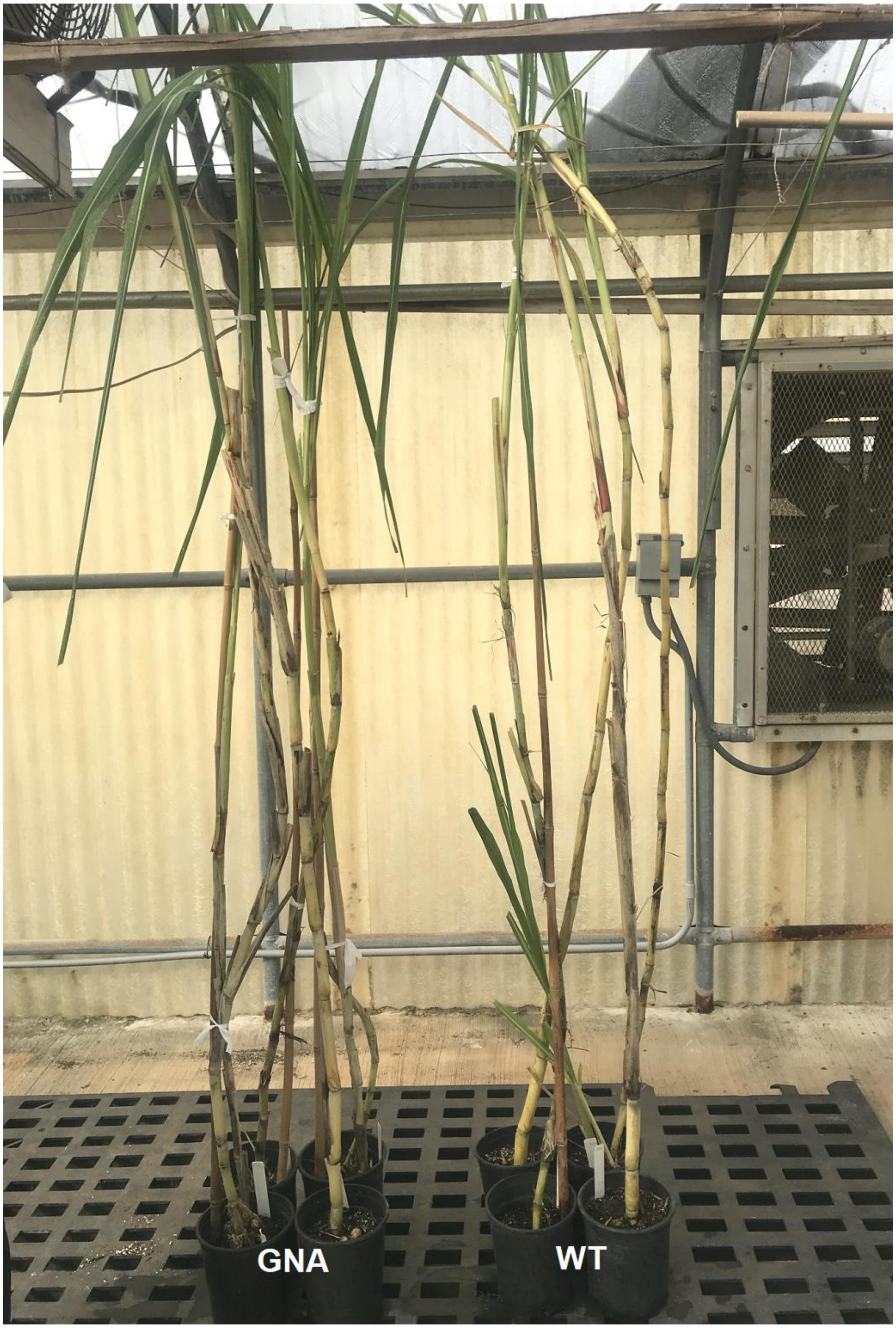

Supplementary Figure S2. Qualitative phenotype of the triple-promoter:*GNA*-expressing sugarcane lines. One-year-old mature plants are shown. GNA: *GNA* transgenic plants; WT: wild type plants.

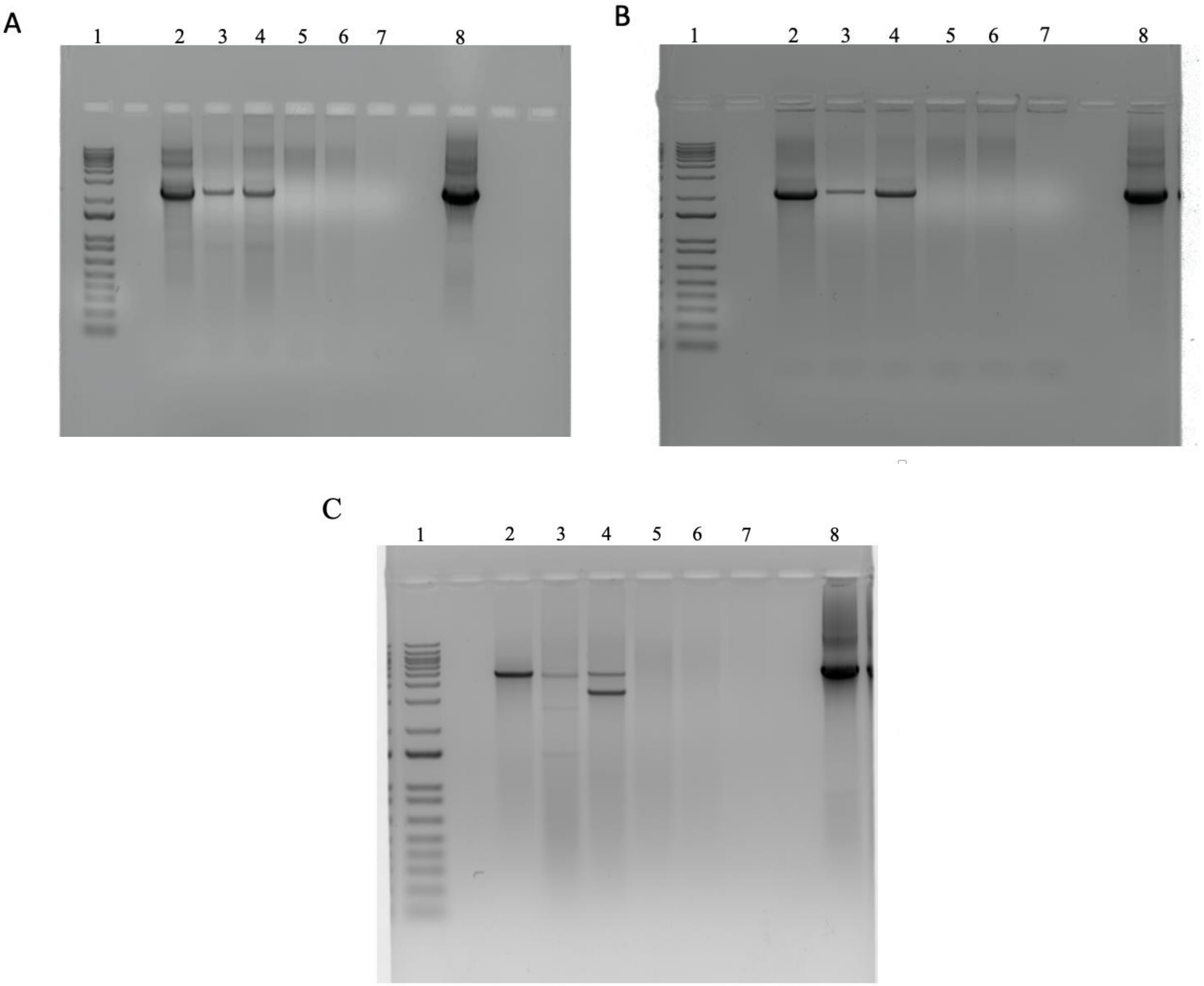

Supplementary Figure S3. (A), (B) and (C) Uncropped agarose gel used to prepare FIGURE 2C. **(A)** shows Integration of *pUbi:GNA* expression cassettes: lane 1, 1 kb DNA plus ladder; lane 2, *pUBD5-1:GNA* line 1-2T; lane 3, *pUBD5-1:GNA* line 5-1G; lane 4, *pUBD5-1:GNA* line 5-3C; lanes 5 and 6, non-transformed (NT) CP72-1210 and CP89-2143, respectively; lane 7, NC-no DNA template and lane 8, *pUbi:GNA* plasmid. **(B)** shows Integration of *pSCBV21:GNA* expression cassettes: lane 1, 1 kb DNA plus ladder; lane 2, *pUBD5-1:GNA* line 1-2T; lane 3 *pUBD5-1:GNA* line 5-1G; lane 4, *pUBD5-1:GNA* 5-3C; lanes 5 and 6, NT CP72-1210 and CP89-2143, respectively; lane 7, NC-no DNA template and lane 8, *pSCBV21:GNA* plasmid. **(C)** shows Integration of *pSHDIR5-1:GNA* expression cassettes: lane 1, 1 kb DNA plus ladder; lane 2, *pUBD5-1:GNA* line 1-2T; lane 3 *pUBD5-1:GNA* line 5-1G; lane 4, *pUBD5-1:GNA* line 5-3C (full and truncated amplicons); lanes 5 and 6, NT CP72-1210 and CP89-2143, respectively; lane 7, NC-no DNA template and lane 8, *pSHDIR5-1:GNA* plasmid.

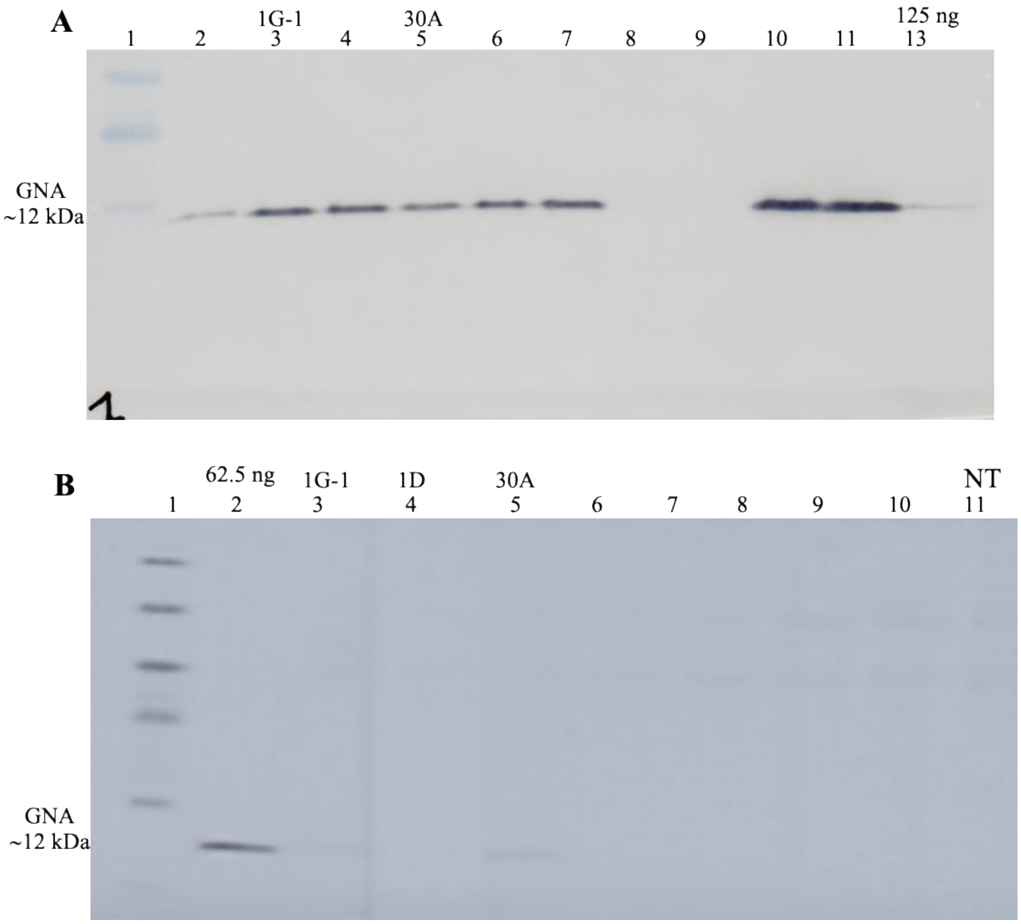

Supplementary Figure S4. (A) and (**B**) Full-length uncropped immunoblot membranes used to prepare FIGURE 1C and 1D. **(A)** shows uncropped immunoblot used to prepare FIGURE 1C (leaves) single-promoter *pUbi:GNA* lines 1G-1: lane 3 and 30A: lane 5 and GNA standard 125 ng: lane 13. **(B)** shows uncropped immunoblot used to prepare FIGURE 1D (culms) single-promoter *pUbi:GNA* lines 1G-1: lane 3, 1D: lane 4 and 30A: lane 5 and non-transformed (NT): lane 11.

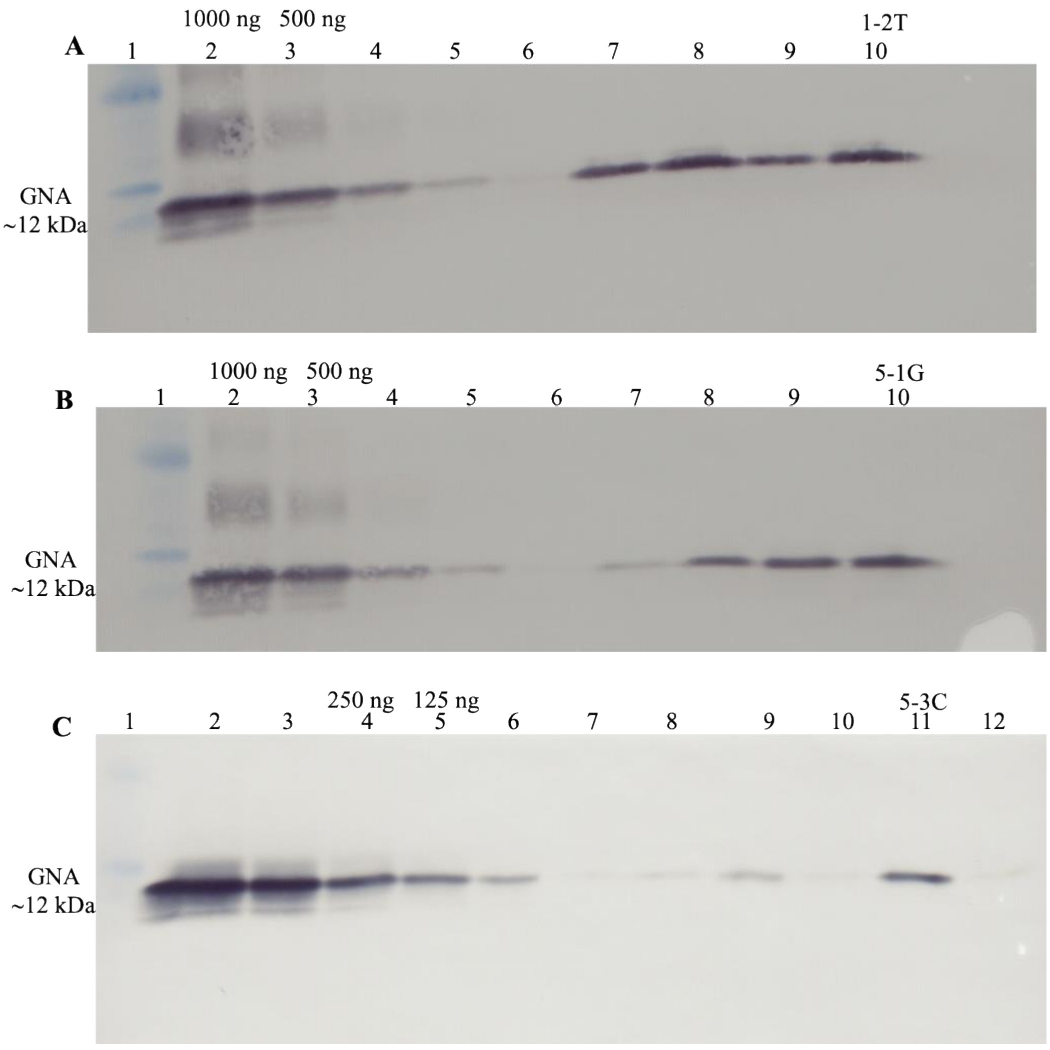

Supplementary Figure S5. (A), (B) and (**C**) Full-length uncropped immunoblot membranes used to prepare FIGURE 2D (Immunoblot for leaf samples). **(A)** shows GNA standard 1000 ng and 500 ng: lanes 2 and 3, respectively, and triple-promoter *pUBD5-1:GNA* line 1-2T: lane 10. **(B)** shows GNA standard 1000 ng and 500 ng: lanes 2 and 3, respectively, and triple-promoter *pUBD5-1:GNA* line 5-1G: lane 10. **(C)** shows GNA standard 250 ng and 125 ng: lanes 4 and 5, respectively, and *pUBD5-1:GNA* line 5-3C: lane 11.

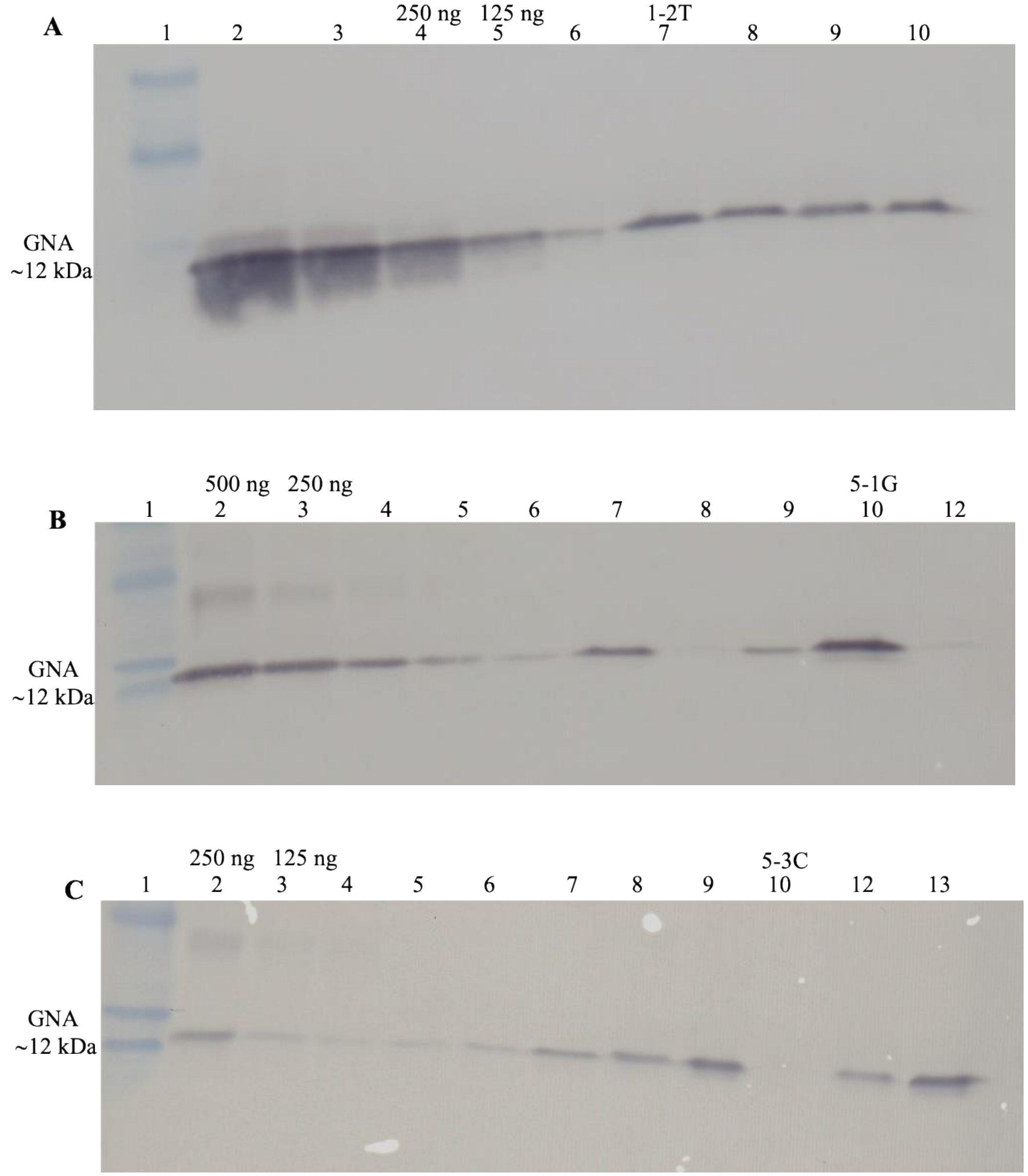

Supplementary Figure S6. (A), (B) and (**C**) Full-length uncropped immunoblot membranes used to prepare FIGURE 2E (Immunoblot for culm samples). **(A)** shows GNA standard 250 ng and 125 ng: lanes 4 and 5, respectively, and triple-promoter *pUBD5-1:GNA* line 1-2T: lane 7. **(B)** shows GNA standard 500 ng and 250 ng: lanes 2 and 3; respectively, triple-promoter *pUBD5-1:GNA* line 5-1G: lane 10. **(C)** shows GNA standard 250 ng and 125 ng: lanes 2 and 3; respectively, and triple-promoter *pUBD5-1:GNA* line 5-3C: lane 10.

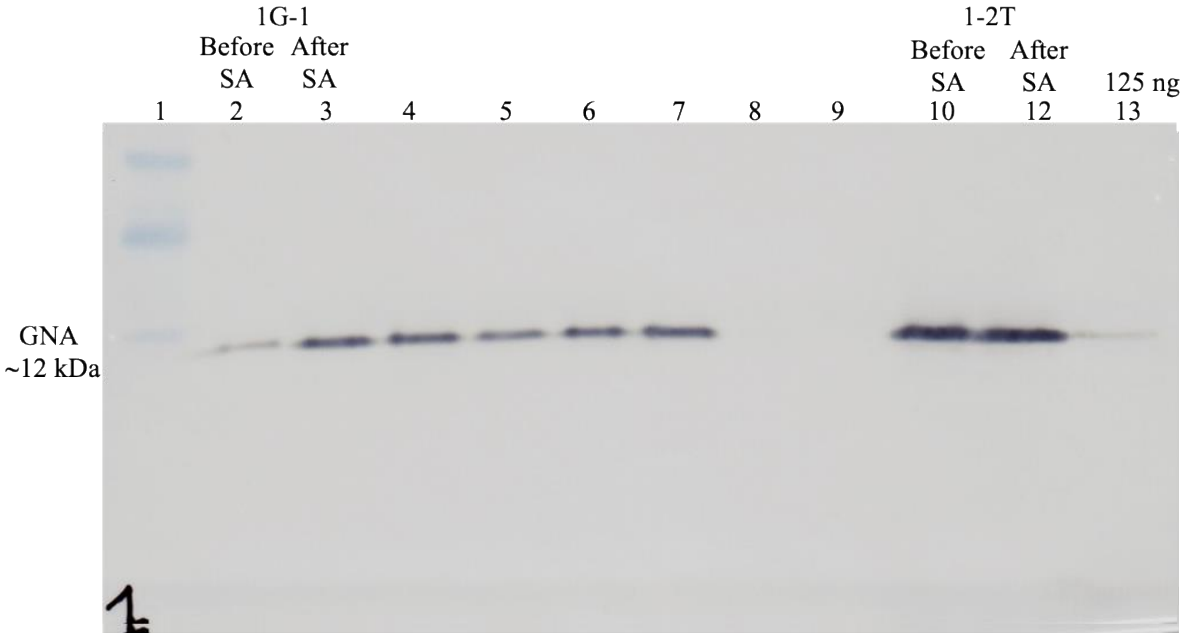

Supplementary Figure S7. Full-length uncropped immunoblot membrane used to prepare FIGURE 3. Lanes 2 and 3 show single-promoter *pUbi:GNA* line 1G-1 before and after salicylic acid treatment, respectively; lanes 10 and 12 show triple-promoter *pUBD5-1:GNA* line 1-2T before and after salicylic acid treatment, respectively; lane 13 shows 125 ng of GNA standard.

Shredding of transgenic sugarcane culms	**Shredded culms**<br>**25 g**
Fine grinding of tissue in<br>IKA®-WERKE M20 Universal Mill	**Grinding**
Extraction buffers:<br>0.1M citric acid/0.2 M sodium acetate buffer (pH 4.0)<br>or<br>0.1M sodium acetate /0.2 acetic acid buffer (pH 5.2),<br>with 1 mM EDTA and 0.05% (v/v) Tween-20	**Extraction**
Solid removal by centrifugation at 12,000 *g* for 20 min, and clarification of supernatant by passing through four layers of Miracloth®	**Extract clarification**
Acetone precipitation and centrifugation at 12,000 *g* for 20 min at 4°C; pellet resuspension in loading dye	**Precipitation and centrifugation**

Supplementary Figure S8. Flowchart of the bench-scale extraction of recombinant GNA from transgenic sugarcane and energy cane culms.

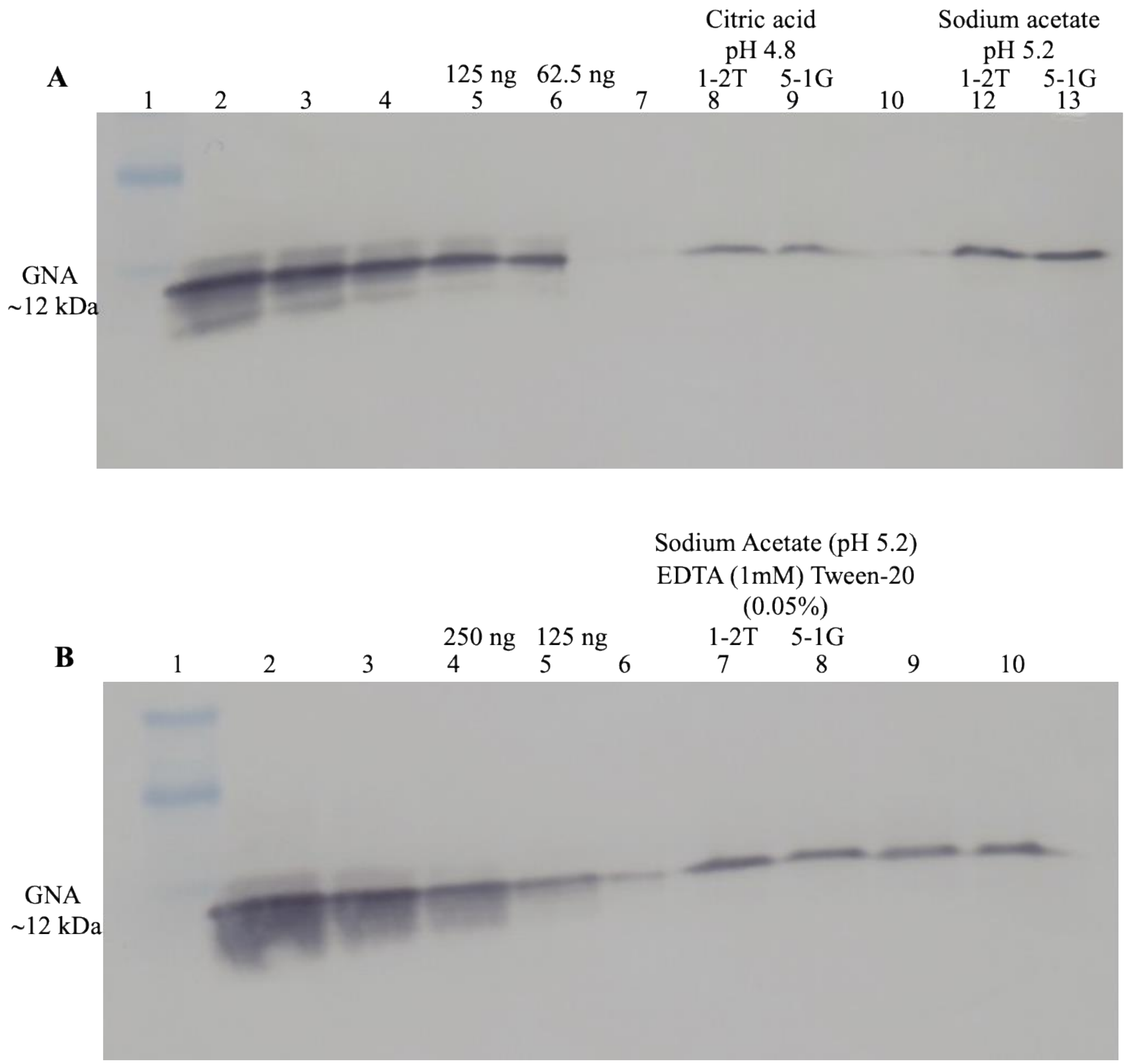

Supplementary Figure S9. (A) and (**B**) Full-length uncropped immunoblot membrane used to prepare FIGURE 4. **(A)** shows the following: GNA standard 125 ng and 62.5 ng: lanes 5 and 6, respectively; triple-promoter *pUBD5-1:GNA* lines 1-2T and 5-1G total soluble protein (TSP) extraction with citric acid pH 4.8: lanes 8 and 9, respectively; TSP extraction of the same lines with sodium acetate pH 5.2: lanes 12 and 13, respectively. **(B)** shows GNA standard 250 ng and 125 ng: lanes 4 and 5, respectively, and triple promoter *pUBD5-1:GNA* lines 1-2T and 5-1G TSP extractions with sodium acetate (pH5.2)/EDTA/Tween-20: lanes 7 and 8.

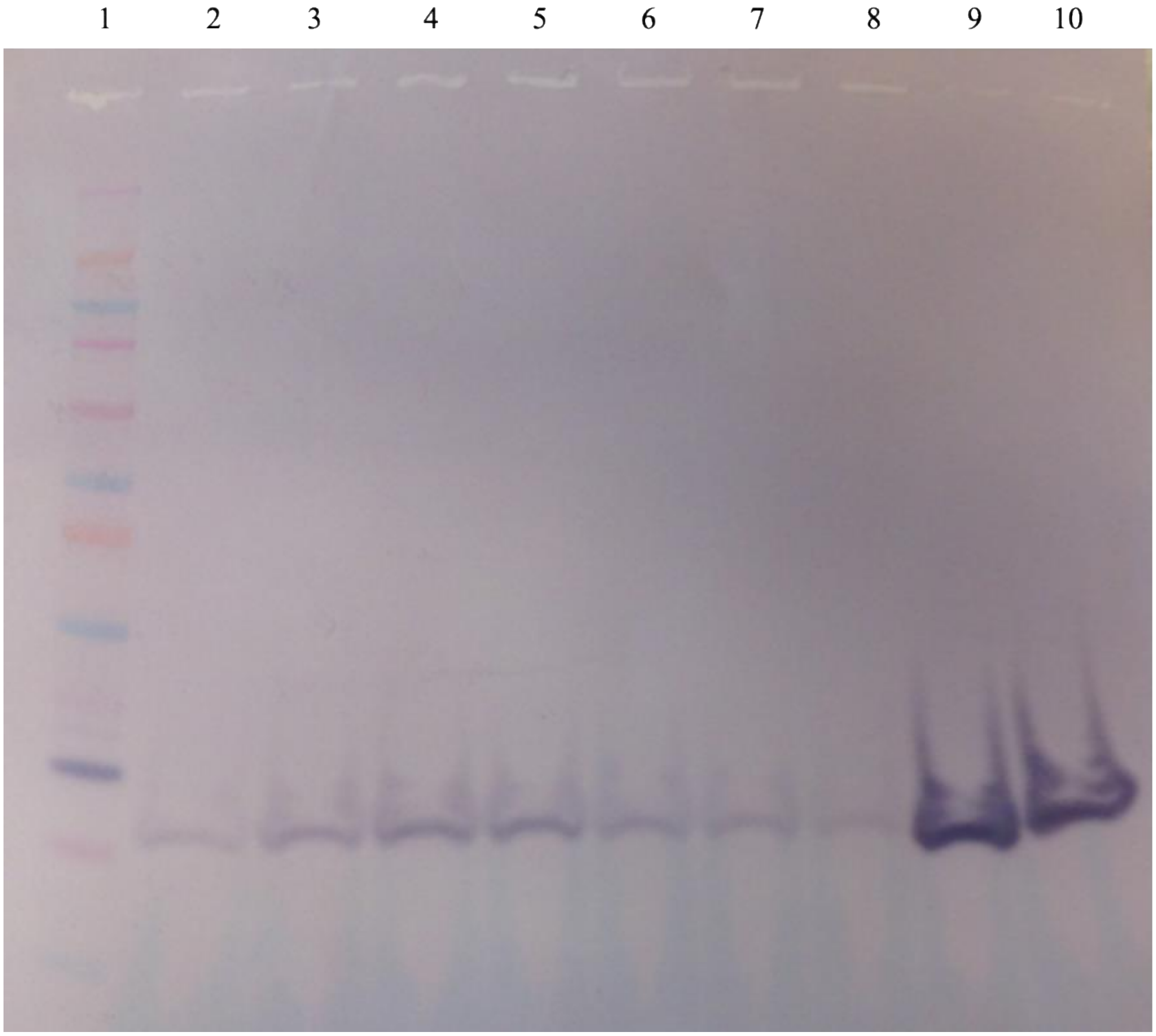

Supplementary Figure S10. Full-length uncropped immunoblot membrane used to prepare FIGURE 5A2. Lanes 1 to 7 represent elution fractions 1.A.7, 1.A.8, 1.A.9, 1.A.10., 1.A.11, 1.A.12 and 1.B.1, respectively.

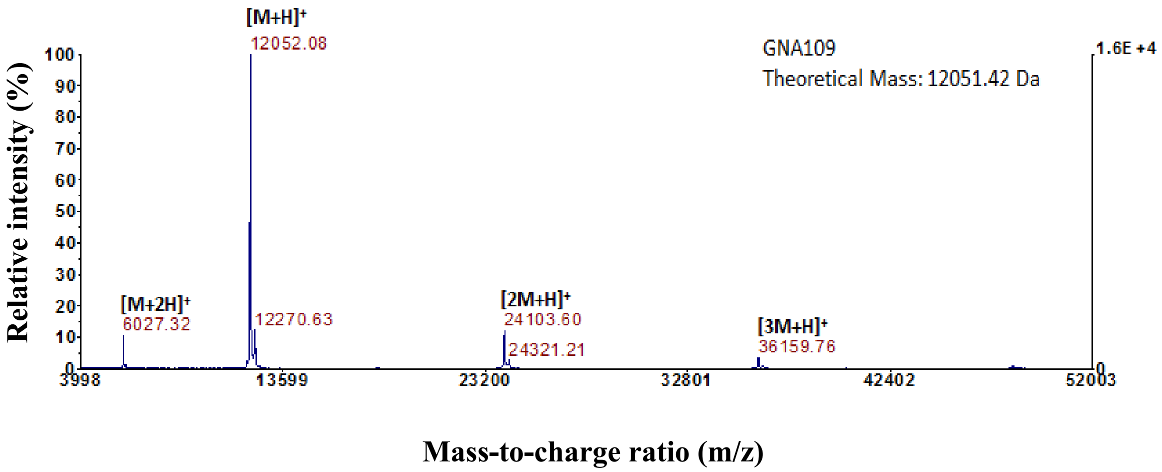

Supplementary Figure S11. Analysis of recombinant GNA_{109} under non-reduced conditions using Matrix-Assisted Laser Desorption Ionization Mass Spectrometry (MALDI-TOF MS). The MS spectra of GNA_{109} display the molecular weights of *m/z* 12,052.08 $[M+H]^+$, *m/z* 24,103.60 $[2M+H]^+$ and *m/z* 36,159.76 $[3M+H]^+$, representing the monomer, dimer and trimer of GNA_{109}, respectively.

```
LEC2GNA    MAKASLLILAAIFLGVITPSCLSDNILYSGETLSTGEFLNYGSFVFIMQEDCNLVLYDVD
1NIV_A     -----------------------DNILYSGETLSTGEFLNYGSFVFIMQEDCNLVLYDVD
                                  *************************************

LEC2GNA    KPIWATNTGGLSRSCFLSMQTDGNLVVYNPSNKPIWASNTGGQNGNYVCILQKDRNVVIY
1NIV_A     KPIWATNTGGLSRSCFLSMQTDGNLVVYNPSNKPIWASNTGGQNGNYVCILQKDRNVVIY
           ************************************************************

LEC2GNA    GTDRWATGTHTGLVGIPASPPSEKYPTAGKIKLVTAK
1NIV_A     GTDRWATGTHTG-------------------------
           ************
```

Supplementary Figure S12. Multiple alignment of amino-acid sequences of the translated GNA peptide transformed into sugarcane (*Galanthus nivalis* lectin; LECGNA2, Protein Bank accession number AAA33346) and the mature snowdrop-bulb native GNA (Vector Labs) (*Galanthus nivalis* agglutinin; 1NIV, Protein Data Bank accession number 1NIV_A). The consensus sequence motif QXDXNXVXY (QEDCNLVLY), involved in recognition of the α-D-mannose substrate, is boxed in red. Multiple amino-acid sequence alignment was performed using ClustalW 2.0 program (www.ebi.ac.uk/clustalw).

Supplementary TABLE S1 Primers used for determination of promoter:*GNA* cassette integration in staked triple promoter:*GNA* sugarcane lines.

**Primer**	**Primer sequence (5’-3’)**
pUbi-F	TGTGCATGTGTTCTCCTTTTT
pSHDIR5-1-F	TGTGGGCGACAGCTTGATAC GTGT
pSCBV21-F	CAGATGCTTGTGCAACTGGT
pSHEF1α-F	CACTTGTTCCCTTGCTGGTT
35ST-R	GCTCAACACATGAGCGAAAC
GNA127-R	TCCCCTGTAGAGAGAGTC
